# A Genomic Reappraisal of Symbiotic Function in the Aphid/*Buchnera* Symbiosis: Reduced Transporter Sets and Variable Membrane Organisations

**DOI:** 10.1371/journal.pone.0029096

**Published:** 2011-12-27

**Authors:** Hubert Charles, Séverine Balmand, Araceli Lamelas, Ludovic Cottret, Vicente Pérez-Brocal, Béatrice Burdin, Amparo Latorre, Gérard Febvay, Stefano Colella, Federica Calevro, Yvan Rahbé

**Affiliations:** 1 UMR203 BF2I, Biologie Fonctionnelle Insectes et Interactions, INSA-Lyon, INRA, Université de Lyon, Villeurbanne, France; 2 BAMBOO, INRIA Rhône-Alpes, Montbonnot Saint-Martin, France; 3 Instituto Cavanilles de Biodiversidad y Biología Evolutiva, Universidad de Valencia, Valencia, Spain; 4 Área de Genómica y Salud, Centro Superior de Investigación en Salud Pública (CSISP), Valencia, Spain; 5 CIBER Epidemiología y Salud Pública (CIBERESP), Madrid, Spain; 6 UMR CNRS 5558 LBBE, Laboratoire Biométrie et Biologie Evolutive, Université Lyon 1, Université de Lyon, Villeurbanne, France; 7 Université de Lyon, Centre Technologique des Microstructures-CTµ, Villeurbanne, France; Technion-Israel Institute of Technology, Israel

## Abstract

*Buchnera aphidicola* is an obligate symbiotic bacterium that sustains the physiology of aphids by complementing their exclusive phloem sap diet. In this study, we reappraised the transport function of different *Buchnera* strains, from the aphids *Acyrthosiphon pisum*, *Schizaphis graminum*, *Baizongia pistaciae* and *Cinara cedri,* using the re-annotation of their transmembrane proteins coupled with an exploration of their metabolic networks. Although metabolic analyses revealed high interdependencies between the host and the bacteria, we demonstrate here that transport in *Buchnera* is assured by low transporter diversity, when compared to free-living bacteria, being mostly based on a few general transporters, some of which probably have lost their substrate specificity. Moreover, in the four strains studied, an astonishing lack of inner-membrane importers was observed. In *Buchnera,* the transport function has been shaped by the distinct selective constraints occurring in the Aphididae lineages. *Buchnera* from *A. pisum* and *S. graminum* have a three-membraned system and similar sets of transporters corresponding to most compound classes. Transmission electronic microscopic observations and confocal microscopic analysis of intracellular pH fields revealed that *Buchnera* does not show any of the typical structures and properties observed in integrated organelles. *Buchnera* from *B. pistaciae* seem to possess a unique double membrane system and has, accordingly, lost all of its outer-membrane integral proteins. Lastly, *Buchnera* from *C. cedri* revealed an extremely poor repertoire of transporters, with almost no ATP-driven active transport left, despite the clear persistence of the ancestral three-membraned system.

## Introduction

All living organisms have been associated with bacteria since the early stages of evolution, generating different degrees of complexity and durablility in these associations varying between pathogenesis and mutualism [1], [2]. In addition to gut bacteria, about 20% of insect species harbour intracellular symbiotic bacteria called endocytobiotes or endosymbionts [3], [4], [5]. Many endosymbionts are associated with insects that live on unbalanced diets and they are essential for the persistence of the hosts in their ecological niches (primary symbionts). This is the case for *Buchnera aphidicola,* the obligate symbiotic intracellular bacteria of aphids, which has been associated for more than 150 million years with these insects [6], providing them with the capability to feed exclusively on the phloem sap of plants.

The association between *Buchnera* (the genus name *Buchnera* is used in this work to design the species name - *Buchnera aphidicola* - when the host strain is not specified) and the primitive aphids has been so successful that almost all members of the Aphididae family are currently harbouring, and strictly co-speciating, with this gamma-proteobacterium [7]. In this work, we analysed more specifically four *Buchnera* strains from the aphids *Acyrthosiphon pisum* (*BAp*), *Schizaphis graminum* (*BSg*), *Baizongia pistaciae* (*BBp*) and *Cinara cedri* (*BCc*), for which the complete genome sequences were available at the beginning of this study [8], [9], [10], [11], [12]. *A. pisum* and *S. graminum* belong to the subfamily Aphidinae and diverged from each other about 50 million years ago [9], [13], whereas *B. pistaciae* and *C. cedri*, from the subfamilies Eriosomatinae and Lachninae respectively, belong to two lineages which are very divergent from the Aphidinae. However, their phylogenetic position is controversial. Thus, in some reconstructions both species cluster together [14] whereas, in other studies, *C. cedri* represents the more basal clade rooting the aphid phylogenetic tree [15].

*A. pisum* and *S. graminum* are both cosmopolitan and oligophagous aphids from temperate climates, thriving on grasses, mainly on a taxonomically restricted set of host plants, *i.e*., Fabaceae and Poaceae respectively [16], [17], [18]. For some aphid populations, apart from *Buchnera,* several secondary symbionts are often coexisting in the same individual; however, these secondary symbionts remain facultative as they are not strictly required for the survival of all aphid species and their prevalence is variable across natural aphid populations [19]. The gall-forming aphid, *B. pistaciae,* is a minor pest species with a complex life cycle that includes a bi-annual alternation of hosts between the pistachio tree, as primary host, and grass roots as the secondary host [18], [20]. Compared to the other three species, *C. cedri* is the most specialised aphid, thriving only on cedar trees. The obligate symbiont *Buchnera,* in *C. cedri,* is permanently associated with “*Candidatus* Serratia symbiotica”, a facultative endosymbiont in other aphid species but a co-primary endosymbiont in this aphid. Thus, both bacteria have established a metabolic complementation for the biosynthesis of tryptophan, *Buchnera* having retained the genes *trp*EG coding for antranilate synthase, the first enzyme of the pathway, whereas the rest of the genes (*trp*ABCD) are located in “*Ca.* Serratia symbiotica” where tryptophan is produced for the whole system [21]. *BCc* bears the most degenerate/incomplete *Buchnera* genome. Overall, these aphids and their associated symbionts may be classified into “successful” (*Ap* and *Sg*) or “less successful” (*Bp* or *Cc*) taxa, both in terms of fitness, *e.g*. the relative growth rates of *A. pisum* and *S. graminum* are three to four times higher than that of a *Cinara* species [22], [23], [24], as well as in terms of adaptive traits (oligophagous versus monophagous).

The *Buchnera* genomes of the four aphid species are all characterized by small size (from 416 kb for *BCc* to 641 kb for *BAp*), a low GC-content (about 25%) and standard bacterial gene density (about 85% of coding DNA). The differences between these four aphid species, as regards the physiology and the biology of the symbiotic interactions, created specific evolutionary constraints contributing to the formation of the different *Buchnera* gene repertoires. The trophic nature of the *Buchnera*/aphid symbiosis has been extensively discussed in the literature (reviewed in: [25], [26]). Experimental studies, using a combination of controlled artificial diets and antibiotic-treated (aposymbiotic) aphids, have focused on the specific aphid requirements of essential amino acids, and some vitamins, not present in the phloem sap. Douglas *et al*. [27] have provided evidence for the synthesis of methionine by *Buchnera* in the aphid *Myzus persicae*, and Douglas and Prosser [28] for that of tryptophan in *BAp.* The involvement of *BAp* in the transfer of nitrogen, from glutamine or glutamate, to other amino acids has been demonstrated by Sasaki and Ishikawa [29]. Febvay *et al*. have provided direct evidence for the biosynthesis of threonine, isoleucine, leucine, valine and phenylalanine by *BAp* [30], [31], highlighting the ability of *BAp* to adapt its production of amino acids to the aphid's nutritional needs [32]. Riboflavin production by *BAp* was also indicated from the results of transcriptomic analyses [33], [34].

More recently, using the knowledge from the genomics of the symbiotic bacteria and their metazoan host, as well as network modelling, the metabolic interdependence of the two partners has been extensively demonstrated [35]. For example, in the biosynthetic pathways of the three essential amino acids leucine, valine and isoleucine, it has been proposed that the last transamination step could occur in the aphid cells, whereas the whole initial pathway is performed by *BAp* [36]. Similarly, Thomas *et al*. [37] revealed a coupling between the histidine and purine biosynthesis pathways, via the partial truncation of the purine biosynthesis pathway, prior to the production of AICAR (5-aminoimidazole-4-carboxamide ribonucleoside). As for transaminations, the sharing of essential pathways between *BAp* and their hosts prevents the bacterium from exploiting the nutrients obtained without reciprocating in some way for the aphid.

From these analyses, it appears that the transport function is a keystone for the persistence of the symbiotic relationships, although the genomic analyses of the four sequenced genomes of *Buchnera* have revealed a very poor repertoire of transporter genes. In this work, we took advantage of several bioinformatic tools to explore and compare the metabolic networks, such as the information system MetExplore [38] and the algorithm PITUFO [39], and to make a systematic analysis of the transport function in *BAp*. The graph-theory based information system, MetExplore, offers the user the possibility of extracting metabolic networks based on genomic annotations, and of reconstructing the corresponding set of pathways through a BioCyc interface [40], [41], as opposed to the KEGG database that performs mapping of the genomics annotation on predefined expert-collated pathways [42]. PITUFO is a graph-theory based algorithm that permits the extraction of precursor sets of compounds (*precursors* being defined as compounds not produced by the network, but necessary for the production of a target metabolite). The use of these tools allowed us to perform a systemic analysis of the input and output compounds (see “Definitions” further on in the Results section) from the *BAp* metabolic network, integrating knowledge about the symbiotic relationships of the system.

In aphids, *Buchnera* are located in the cytosol of specialized insect cells called bacteriocytes. Within bacteriocytes, *Buchnera* are housed inside symbiosomal vesicles whose role remains largely unknown but that are probably playing an important role in the regulation of trophic exchanges. The second aim of our study was to characterise these vesicles, whose precise organisation and functions still needed to be elucidated. Histological analyses revealed that these vesicles show a characteristic three-membraned structure similar to that described in many, though not all, insect endosymbioses [43], [44]. Although the evolutionary routes between *Buchnera* and mitochondria are different (e.g., the integration process of *Buchnera* did not include gene transfer to the host), a structural/functional comparison of the symbiosome and the mitochondria was carried out in this paper as convergent solutions might have been selected independently during both evolutionary processes. In particular, we tried to detect whether extensive contact points are preserved in the three-membraned symbiotic structures as these are thought to represent important transport systems in mitochondria [45], such as the polypeptide import machinery (the so-called TIM-TOM complex) or the cross-membrane transport system for nucleotides (kinases voltage-dependent anion channel/adenine nucleotide translocator - VDAC/ANT - complexes [46]). We also analysed potential pH gradients within the symbiosomes that could fuel the active transport of molecules between the cytoplasm of the bacteriocyte and the bacterial cytosol.

Finally, we also addressed the question of the potential role of the GroEL protein in the *Buchnera* transport system, as well as the hypothetical transport of GroEL itself in the aphid compartment. Although GroEL is the most highly expressed, and the first detected, protein in aphid symbionts, its role has long been a matter of conjecture. The most popular idea, consistent with it being one of the most highly conserved proteins in *Buchnera*, is that it acts as a non-specific folding auxiliary to *Buchnera*'s nascent proteins, which suffer from a general accumulation of mildly deleterious mutations with associated thermodynamic instability [47]. However, because of its involvement in plant virus protection in the hemolymphatic stream from the digestive tract to the salivary cells [48], and/or its presence as a glycosylated entity in the aphid digestive epithelium [49], it has been suggested that GroEL could have other roles that are more compatible with an exported protein. To test whether the export-related observations could be indicative of a significant export of the protein from the bacteriocytes, we analysed the subcellular location of GroEL within young adult maternal bacteriocytes using immunogold labelling.

In this study, integrating the results from genomic re-annotations of membrane-associated proteins with metabolic network analysis and structural analysis of the symbiosomal membrane system, we have described the transport capabilities of *BAp* and explored how the transport function has been shaped in *Buchnera* by the evolutionary forces, during intracellular evolution (especially genome shrinkage), in the light of the diversity offered by different host aphids characterised by distinct ecological traits.

## Materials and Methods

### Genomic and metabolic network analyses

#### Genome sequences

The genome sequences of the four *Buchnera* strains used in this work were obtained from GenBank: *BAp,* BA000003 [8]; *BSg*, AE013218 [9]; *BBp*, AE016826 [10]; *BCc*, CP000263 [11]. Two supplementary *Buchnera* strains from *A. pisum* [12] were also used (*BAp*_Tu7, CP001158 and *BAp*_str5, CP001161) in order to differentiate true pseudogenes from potential sequencing errors. The genomic sequence of *Buchnera* from the aphid *Cinara tujafilina* (*BCt*, CP001817) was very recently obtained [50]. However, this sequence was not included in our analysis, although a short comparison of the transport function in *BCc* and *BCt* is given in the Discussion section. The pea aphid genome sequence is available from the NCBI with genome project ID 13657 [16].

#### Transporter identification in *Buchnera* genomes

In this work, transporter family names always follow the official family name designated by **TCDB**, the Transport Classification DataBase (http://www.tcdb.org/). A first run of transporter identification was carried out, through keyword and gene ontology queries, of the four available *Buchnera* genomes using the Swissprot HAMAP (http://www.expasy.org/sprot/hamap/) and TIGR CMR (http://cmr.jcvi.org/) databases.

To complete this analysis, all the retrieved genes and all the *Buchnera* membrane proteins (transmembrane keyword of HAMAP-annotated proteins, *i.e.* harbouring more than 2 transmembrane regions, as predicted by the TMHMM software [51], [52]) were further submitted to a TC-Blast analysis [53]. This allowed us to assess the transport-classification positioning of selected proteins and to evaluate the predictions of their potential substrates (Blast used with no low-complexity filtering). We considered here that predictions associated with Blast expectation values above 10^−40^ (power of 10 given in Table 1) should be treated cautiously, at least in terms of the transported substrate.

**Table 1:** Transport-related genome content of the four *Buchnera* strains *BAp*, *BSg*, *BBp* and *BCc.*

	TCDB links and hits							Genes and UniProt links			
Cl.^a^	Super-family^a^	TC#^b^ of best hit	TC ID^b^ of best hit	exp hit	Substrate [Import-Export]	Quater. structure	TM (i/o)^c^	*Buchnera-A. pisum* d	*Buchnera-S. graminum*	*Buchnera- B. pistaciae*	*Buchnera- C. cedri*
1	MIP/aquaporin	1.A.8.1.1	POAER0	-63	glycerol, water, small uncharged compounds [I-E]	Homo tetramer	8 (i)	GlpF:BU306	GlpF:BUSg_296	-	-
1	MscS	1.A.23.2.1	POCOS1	-99	ions (mechanosensi-tive) [I-E]	Homo-heptamer	3 (i)	YggB:BU452	YggB:BUSg_437	YggB:BBp_402	YggB:BCc_280
1	CC-HSP70	1.A.33.1.2	P0A6Y8	-∞	Nascent prot. Chaperone [I]	Monomer	1 (i)	DnaK:BU153	DnaK:BUSg_146	DnaK:BBp_142	DnaK:BCc_096
1	porin GBP	1.B.1.1.6	Q56828	-75	small solutes [I-E]	Homotrimer	17 (o)	OmpF:BU359	OmpF:BUSg_347	-	-
1	porin OOP	1.B.6.1.1	P0A910	-59	small solutes [I-E]	Homo-dimer	8 (o)	OmpA:BU332	OmpA:BUSg_320	-	OmpA:BCc_210
1	porin OmpIP	1.B.33.1.3	P0A943	-106	proteins [E]	Monomer	1 (o)	YaeT:BU237	YaetT:BUSg_231	-	YaeT:BCc_146
1	CFCV	(1.C.31)	ns hit	-	nd [E]	nd	5 (i)	ΨCvpA:BU168	CvpA:BUSg_162	CvpA: BBp_158	-
2	MFS	2.A.1.2.8	P39843	-17	multidrug [E]	Homodimer	11 (i)	YajR:BU466	YajR:BUSg_450	YajR:BBp_411	-
2	MFS	2.A.1.3.2	P0AEJ0	-5	multidrug/sugar [E]	Monomer	12 (i)	TsgA:BU535	TsgA:BUSg_516	YhfC:BBp_477	-
2	MFS	2.A.1.5.1	P02920	-2	sugar [I-E]	Homodimer	9 (i)	-	YgjT: BUSg_160	YgjT: BBp_156	-
2	MFS	2.A.1.36.1	P43531	-116	multidrug/sugar [E]	Monomer	12 (i)	YnfM:BU588	YnfM:BUSg_567	YnfM:BBp_532	-
2	MFS	2.A.1.38.1	P24077	-3	siderophore, aa [E]	Homodimer	6 (i)	YabI:BU139	YabI:BUSg_132	YabI:BBp_130	-
2	DMT	2.A.7.3.2	P31125	-9	amino acids [E]	Monomer	10 (i)	PagO:BU281	PagO:BUSg_270	Ψ Yeda	-
2	Oxa1 IMP	2.A.9.3.1	P25714	-∞	preprotein translocase	Homodimer	4 (i)	OxaA:BU015	YidC:BUSg_016	YidC:BBp_016	YidC:BCc_007
2	PiT	2.A.20.1.1	P0AFJ7	-167	PO_4_^3-^ [I]	nd	10 (i)	PitA:BU587	PitA:BUSg_566	PitA:BBp_531	-
2	MOP flippase	2.A.66.1.3	P37340	-111	multidrug [E]-Na^+^ [I]	nd	12 (i)	-	-	NorM:BBp_106	-
2	MOP flippase	2.A.66.4.1	P37169	-143	peptidoglycan lipids [E]	nd	13 (i)	MviN:BU333	MviN:BUSg_321	MviN:BBp_309	-
2	PerM permease	2.A.86.1.1	P0AFI9	-7	Small solute [E]	nd	7 (i)	YdiK:BU123	YdiK:BUSg_115	YdiK:BBp_117	-
2	NAAT	2.A.95.1.1	Q8J305	-30	Neutral amino acids [I]	nd	6 (i)	YchE:BU267	YchE:BUSg_257	YchE:BBp_248	-
2	NAAT	2.A.95.1.1	Q8J305	-21	Neutral amino acids [I]	nd	6 (i)	YhgN:BU449	YhgN:BUSg_434	YhgN:BBp_399	-
3	ABC	3.A.1.15.53.A.1.15.53.A.1.15.5	P0A9X1P39832P39172	-78-77-55	Zn^2+^ [I]	ABC membrane binding-p	0 (c)7 (i)0 (c)	ZnuC:BU318ZnuB:BU317-	ZnuC:BUSg_308ZnuB:BUSg_307ZnuA:BUSg_309	ZnuC:BBp_295ZnuB:BBp_294-	---
3	ABC	3.A.1.106.1	P60752	-62-65	multidrug-like, lipids [E]	ABC & membrane binding-p	6 (i)	MdlA:BU479/MdlB:BU480-	MdlA:BUSg_464/MdlB: BUSg_465-	MdlA:BBp_423/MdlB:BBp_424-	MdlA:BCc_297/MdlB:BCc_298-
3	ABC	3.A.1.121.2	O54396	-63	ATP-binding protein	ABC membrane binding-p	0 (c)--	Uup:BU364--	Uup: BUsg_352--	---	---
3	ABC	3.A.1.125.13.A.1.125.13.A.1.125.1	P75957P75956P75958	-61-74-54	lipoprotein [E]	ABC membrane binding-p	0 (c)4 (i)0	LolD:BU296LolC:BU295,Ψ LolE:BU297	LolD:BUSg_285LolC:BUSg_284ΨLolE: BUSg_286	---	---
3	F-ATPase	3.A.2.1.1	8 proteins	-	H^+^ [I-E]	Multimeric complex	nd (i)	AtpA-H:BU002-BU009 (8)^e^	AtpA-H: BUSg_002-009 (8)	AtpA-H: BBp_00 2-009 (8)	-(0)
3	e^-^ trsp	3.D.6.1.1	7 proteins	-	electrons, ions [I-E]	Multimeric complex	nd (i)	RnfABCDEG(6)	RnfABCDEG(6)	RnfABCDEG(6)	-
3	Type II SP	3.A.5.1.1	11 proteins	-	protein [E]	Multimeric complex	nd (i)	Ffh, LepAB, SecABGEY, YajC, LspA, Tig (11)	Ffh, LepAB, SecABGEY, YajC, LspA, Tig(11)	Ffh, LepAB, SecAGEY, YajC,LspA(9)	Ffh, LepAB, SecAGEY(7)
3	Type III SP	3.A.6.2.1	33 proteins(16+4+13)	-	protein [E]	Multimeric complex	nd(i, o)	FliE-KM-R, FlhAB, FlgA-JKN (14+2+12)	FliE-KM-R, FlhAB, FlgA-JKN(14+2+12)	fliE-KM-R, flhAB, flgBCF-J(14+2+7)	fliF-IN-R, flhAB, flgFHI(10+2+3)
4	PTS-GG	4.A.1.1.14.A.1.1.1	P69783P69786	-52-∞	glucose [I]	Multimeric complex (EIIA/CB)	0 (c)11 (i)	Crr:BU063PtsG:BU356	Crr :BUSg_060PtsG:BUSg_344	Crr:BBp_059PtsG:BBp_326	--
4	PTS-FM	4.A.2.1.2	P00550	-∞	mannitol [I]	Homo-dimer (EIICBA)	8 (i)	MtlA:BU572	MtlA:BUSg_552	MtlA:BBp_517	-
8	PTS-EI	8.A.7.1.1	P08839	-∞	glucose/mannitol PTS energy coupling Prot	Homodimer	0 (c)	PtsI:BU064	PtsI:BUsg_061	PtsI:BBp_060	-
8	PTS-HPr	8.A.8.1.1	P0AA04	-31	Glucose/mannitol PTS energy coupling Prot	Monomer	0 (c)	PtsH:BU065	PtsH:BUsg_062	PtsH:BBp_061	-
9	HCC	9.A.40.1.2	P0A2L3	-112	Mg^2+^ Co^2+^ [E]	nd	0 (nd)	YbeX:BU443	YbeX:BUSg_428	CorC:BBp_394	-
9	HCC	9.A.40.1.2	P0A2L3	-24	Mg^2+^ Co^2+^ [E]	nd	7 (i)	YoaE:BU323	YoaE:BUSg_314	YoaE:BBp_300	YoaE:BCc_201
-	ns hit	ns hit	ns hit	-	Nascent prot. (chaperone) [I]	Homo-heptamer	0 (c)	GroES:BU018	GroES:BUSg_018	GroES:BBp_020	GroES:BCc_010
-	ns hit	ns hit	ns hit	-	Nascent prot. (chaperone) [I]	2-ring-heptamer	0 (c)	GroEL:BU 019	GroEL:BUSg_019	GroEL:BBp_021	GroEL:BCc_011
-	ns hit	ns hit	ns hit	-	Peptidoglycan binding protein	Monomer	0 (o)	-	-	Pal:BBp_282	-
-	ns hit	ns hit	ns hit	-	protein processing	Homodimer	4 (i)	HtpX: BU321	HtpX:BUSg_313	HtpX:BBp_299	HtpX: BCc_200
-	ns hit	ns hit	ns hit	-	Cl^-^ channel [nd]	nd	3 (i)	YqhA:BUPL02	YqhA: BUSg_pl2	YqhA:BBp_601	-
-	ns hit	ns hit	ns hit	-	unknown	nd	6 (i)	YciC:BU276	YciC:BUSg_265	YciC: BBp_256	YciC: BCc_173
-	ns hit	ns hit	ns hit	-	unknown	nd	1 (nd)	YfgM:BU608	YfgM:BUSg_583	YfgM:BBp_550	YfgM:BCc_397
# genes (% of CDS in the genome)								90 (16%)	92 (17%)	79 (16%)	35 (10%)
# identified transporter systems								33	34	30	12

a : TCDB nomenclature (Transporter Classification Database): 1, Channels/Pores; 2, Electrochemical Potential-driven Transporters; 3, Primary Active Transporters; 4, Group Translocators; 8. Accessory Factors Involved in Transport; 9, Incompletely Characterized Transport Systems.

b : TCDB identification of best hit (from *E.coli* or Enterobacteriaceae if available) with TC-Blast (BLAST expectation values are given in the *exp hit* column).

c : Number of transmembrane domains predicted by HMMTOP [51] for alpha-helix and UniProt protein region description for transmembrane beta-strands (localisation: i, inner membrane; o, outer membrane; c, cytosolic; nd, not determined).

d : Official Swiss-Prot *Buchnera* gene name (or *E. coli* closest homolog if no name given in the primary annotation).

e : number of genes in the complex. Ψ: pseudogene, ns hit: not significant hit.

A synteny/orthology analysis was then performed, using the TIGR CMR genome visualisation tool, to verify the absence of individual missing genes in each *Buchnera* genome and to identify potential pseudogenes. The specialized databases ABCdb [54] and OPM [55] were also used to retrieve specific information on ABC transporters and to infer further functional or structural properties (e.g., substrate specificities, complex partnerships or membrane topology). BuchneraBASE [56] was also used to access the annotations of orthologous genes in *Escherichia coli* (notably from EchoBase and EcoCyc).

#### Comparative genomics

A general comparative genomic analysis was performed using the “Compare Organisms” tool from the TransportDB database (http://www.membranetransport.org/), describing 184 bacteria covering a wide range of the overall bacterial diversity [57]. Statistical analyses were then performed using the R software (http://cran.r-project.org/).

#### Metabolic network analysis (input/output compounds and pathway analysis)

This part of the work was performed on *BAp*. Indeed, genomic information was available only for the host of this strain and, as the overall process requires several manual steps and human expertise, especially for the detection of false positives, analysis of the three other *Buchnera* strains was not possible.

The compound graph, where nodes are compounds and edges are reactions, was built using enzymatic annotations from the AcypiCyc Database [41] and has been extracted for our work from the MetExplore server [38]. Within the compound graph, the automatic identification of subgraphs without incoming edge (i.e., using as entrance a compound that is not produced by any reaction) enabled us to identify the groups of metabolites that are the sources of the networks (that are not reachable from other metabolites) [58]. These compounds are called input compounds. Conversely, an output compound is defined as any compound that is produced by a reaction but is not used by another one in the same organism.

Automated pathway analysis (graph traversal) was performed using the *mapfinder* function of MetExplore [38], producing primary lists of input and output compounds from the metabolic network of *BAp*. The list of input compounds of *BAp* was manually completed by adding all the small and over-abundant compounds filtered from the network to prevent bias in the topology analyses [38]. The list of output compounds was also manually completed as, in fact, any produced compound might by exported by *BAp,* whether it is consumed or not by a reaction within the bacterium. Hence, manual inclusions were made using knowledge from the literature concerning the host requirements (especially for amino acids and vitamins).

Finally, both lists were manually corrected using the PITUFO algorithm [39] and the AcypiCyc database [41]. The PITUFO algorithm calculates, from a given target metabolite and the list of inputs, all the alternative sets of inputs that result in the production of the target metabolite. PITUFO was run only on amino acids and it provided a better understanding of the various interactions between the different amino acid biosynthetic pathways. The AcypiCyc database harbours the metabolic network of several organisms, including *A. pisum* and *Buchnera*. As MetExplore is directly linked to AcypiCyc, the user can easily place any compound or visualise any reaction in a pathway. This global manual refinement was necessary as many false positives were detected by MetExplore due to, for instance, enzyme assignment errors, reversible reactions or reactions that do not occur in *BAp*.

### Electron and confocal microscopy

#### Transmission electron microscopy

Bacteriocytes from young adults of *A. pisum* (clone LL01), were dissected directly in 3% glutaraldehyde, further fixed in the same solution for 2 h, and then post-fixed in 1% osmium tetroxide for 1 h. Younger maternal bacteriocytes were also observed from 3^rd^ instar nymphal *A. pisum*, for which nymphs were decapitated directly in 3% glutaraldehyde, fixed for 2 h 30, and then post-fixed for 17 h in 2% osmium tetroxide, followed by standard preparation and inclusion protocols. Sections were then contrasted in 3% uranyl acetate, followed by lead citrate stain. Basically, the protocol was adapted from the one used for aphid digestive tract observations [59]. In *A. pisum*, ultrastructural observations are only reported for maternal bacteriocytes located in the body cavity. For *B. pistaciae*, females dissected out from pistachio galls were used, as described above for *A. pisum* nymphs. This aphid species is smaller and waxier as compared to others analysed in this work. Therefore the insect fixation was probably a limiting factor of quality that we were not able to fully control for this species. As for *C. cedri*, young reproductive females were used and electron microscopy protocols were followed according to those already published [60]. Image analyses (Fast Fourier Transformation filtering, pixel averaging and automated counting of membranes) were performed using the MacBiophotonics Image J Software [61].

#### Immunogold labelling

Bacteriocytes from young adults of *A. pisum* were dissected in buffer A (25 mM KCL, 10 mM MgCl_2_, 250 mM sucrose, 35 mM Tris-HCl, 1 ‰ diethylpyrocarbonate, pH 7.5), and fixed for 48 h at 4°C in 3% paraformaldehyde, 0.5% glutaraldehyde and 0.1 M phosphate buffer, pH 7. Bacteriocytes were then washed/centrifuged several times in 0.2 M phosphate buffer at 4°C, and immobilized by adding a few drops of melted 1.5% agar on the pellet. The agar block was partially dehydrated in a graded ethanol series. Ethanol (70%) was then progressively replaced with LR White (London Resin, cat#14380) embedding medium, by polymerization of the resin at 60°C under oxygen-free conditions. Ultrathin sections (<90 nm) were prepared, using a Reichert Ultracut S ultramicrotome, and deposited on 200 mesh Formvar-coated nickel grids. After blocking the non-specific binding sites, using 1% goat IgG in Tris buffer for 15 min, sections were incubated for 1 h with either a rabbit anti-GroEL serum (gift from the late Prof. Ishikawa) or a rabbit non-immune serum as a negative control, both diluted to 1/1000 in Tris buffer. Grids were then washed in Tris buffer and treated for 1 h with an anti-rabbit 10 nm gold conjugate (Sigma G-7402), diluted 1/50 in 0.5 M NaCl. After a Tris-buffer wash, followed by double distilled water, samples were stained with 4% fresh aqueous uranyl acetate for 20 min in the dark, and finally washed in double distilled water. Grids were examined in a Philips CM120 transmission electron microscope. Except for the uranyl acetate, all solutions used were filtrated through a 0.22 µm membrane.

#### Confocal microscopy

We used confocal microscopy on *ex-vivo* bacteriocytes, incubated with pH-sensitive SNARF® AM acetate ester probes (trademark dye pH indicator by Molecular Probes Inc.) to detect intracellular pH gradients within these individual *Buchnera*-harbouring host cells. Upon incubation, the membrane-permeating probe is cleaved by endogenous cell esterases and the soluble pH-sensitive fluorophore is trapped in the corresponding intracellular compartment [62] including, in this case, *Buchnera*. The detailed protocol is as follows. Bacteriocytes were dissected out from young adult of *A. pisum* using an aphid-specific iso-osmotic buffer [63]. They were then incubated for 45 min, at 25°C, in the presence of 5 mM carboxySNARF®-1 acetomethyl ester acetate [64] purchased from Molecular Probes. Bacteriocytes were observed under a Zeiss LSM510 META confocal microscope (x63 objective, zoom 5, pinhole 1.18 Airy Unit) with 488 nm excitation and a 585 nm/640 nm emission set-up, specifically for ratiometric analyses [62]. The meta (spectral) mode was also used when applicable, and to ensure that probe intracellular penetration was effective; intracellular ROI levels (region of interest) were used to check *in situ* SNARF® emission spectra and were positioned, through the Zeiss LSM-510 software, either within the *Buchnera* cytoplasm, or within bacteriocyte nuclei, or within bacteriocyte bacteria-free cytoplasmic fields. Image ratios were calculated as simple weighted pixel ratio with offset (585/640).

## Results

### Reappraisal of the transport capabilities of *Buchnera*

Based on the genomic annotations and expertise from the Transporter Classification DataBase (TCDB, see Methods), the transport capabilities of the four *Buchnera* strains have been summarized in Table 1 (including links to database resources).

Although all *Buchnera* genes involved in transport have an ortholog in *E. coli*, manual refinement of the TCDB automated annotation for *Buchnera* enabled us to describe about 20 supplementary transport systems, as compared to the 14 automatically detected. The transport systems automatically detected were: 4 ATP-binding Cassette (ABC), 1 F-ATP synthase, 1 Major Intrinsic Protein (MIP), 5 Phosphotransfer-driven group Translocators Superfamily (PTS), 2 out of 5 Major Facilitator Superfamily (MFS) and 1 Inorganic Phosphate Transporter (Pit). The *Buchnera* transport systems referenced in TCDB and found in *E. coli,* although not automatically detected, were: 1 (partially described in *E. coli*) secretion system, 1 Small Conductance Mechanosensitive Ion Channel (MscS), 3 out of 5 MFS, 1 Drug/Metabolite Transporter (DMT), 1 Cytochrome Oxidase Biogenesis (Oxa1) and 2 Multidrug/Oligosaccharidyl-lipid/Polysaccharide (MOP) flippase. The following transport systems were not automatically described in *Buchnera* or in *E. coli,* although referenced in the database with annotations from very closely related bacteria, such as *Salmonella*: 1 T3-secretion system, 1 electron transport chain, 1 HlyC/CorC (HCC), 1 GroESL and 1 DnaK Heat Shock Proteins (HSP), 3 porins, 1 Autoinducer-2 Exporter permease (PerM) and 2 Neutral Amino Acid Transporter (NAAT). Finally, 4 hypothetical proteins with significant transmembrane domains produced no hit in the TCDB database.

To summarize, *BAp* and *BSg* possess 33 and 34 identified transport systems encoded by 90 and 92 genes, representing 16 and 17% of the coding sequences (CDS) of their genome respectively. *BBp* shows 30 transport systems encoded by 79 genes (16% of the genome) and *BCc* reveals only 12 transport systems encoded by 35 genes, representing only 10% of its genome. The transporters identified in this work are described below, according to the TCDB classification [57]. It is to note that substrate specificity remains very speculative for 10 genes in *BAp*, as well as for the corresponding orthologs in *BSg*, *BBp* and *BCc* (*yajR, tsgA, ynfM, ybaI, pagO, ydiK, ychE, yhgN, yoaE and yqhA).* These genes and their transporter activities are retained here as hypothetical interesting candidates for future functional studies in *Buchnera*.

#### Class 1. Channels/pores

The four strains of *Buchnera* possess the small-conductance mechanosensitive ion channel (MscS encoded by *yggB*) that mediates the opening, in response to stretch forces, of the membrane lipid bilayer without the need for other proteins. This transporter is a general ion channel with a general preference for anions, as described for *E. coli* [65]. It is reported to be involved in osmotic pressure regulation in bacteria.

*BAp* and *BSg* possess a MIP/aquaporin (encoded by *glpF*) known as a glycerol and small uncharged compound (water, NH3) transporter. The MIP is absent from *BBp* and *BCc*. The general bacterial porins (GBPs), encoded by *ompF* and *ompA,* are present in *BAp* and *BSg*, whereas *BCc* possesses only OmpA. *BBp* has lost both OmpA and OmpF and preserved the Pal protein, which is not membrane-spanning but bears a highly conserved OmpA-like domain (see Discussion). OmpF is a general outer-membrane porin involved in the exchanges of small solutes and in shape stabilization of the bacterium, whereas the role of OmpA remains unclear in *E. coli*. The OmpIP protein (encoded by *yaeT* and named BamA in *E. coli*) is present in *BAp*, *BSg* and *BCc* (but absent in *BBp*). In *BAp*, *yaeT* was described as a putative pseudogene (truncated and fused with *fabZ*) in BuchneraBASE [56]. However, the gene is present, and not truncated, in the two strains of *BAp* _Tu7 and *BAp* _str5 newly sequenced by Moran *et al.* [12]. Hence, the truncation is probably a sequencing error or a sequence specificity of the *BAp* clone (named Tokyo strain), sequenced by Shigenobu *et al*. [8], but not generally relevant to all *A. pisum Buchnera* strains. In *E. coli*, this protein, that is not a porin despite its TCDB classification, is involved with BamB, BamC, BamD and BamE in the export and scaffolding of outer-membrane proteins and, therefore, is a key component of outer membrane biogenesis and stability [66]. It should be noted that BamD and BamE are present in *BAp*, *BSg* and *BCc,* although BamB and BamC have been lost in these organisms. *BBp* has lost all of the members of the Bam family.

The colicin V production protein (CvpA) is an inner-membrane protein required for the production of the microcin/colicin V toxin in *E. coli* [67]. It is only present in *BBp,* where its role remains unknown. It is important to note that CvpA shares domain homology with the LysE family (InterPro:IPR003825) involved in the efflux of several amino acids (lysine, arginine, threonine and homoserine). CvpA is absent from *BCc* and is present as a pseudogene in *BSg* and *BAp*, as well as in *BAp*_Tu7 and *BAp*_str5 with a single deletion conserved in the three clones from *A. pisum*, while a different deletion is observed in the *BSg* strain.

#### Class 2. Electrochemical potential-driven transporters

While *E. coli* possesses more than 70 genes encoding transporters from the Major Facilitator Superfamily (MFS), which are mostly multidrug exporters, only five are found in *BSg* and *BBp*, four in *BAp* and none in *BCc*. YgjT might be involved in the import/export of sugars (absent in *BAp* and *BCc*), TsgA is possibly involved in sugar export and YabI in the export of siderophore and amino acids. All these transporters are putative candidates for the import/export of some metabolites that shuttle between the associated partners. It is noteworthy that, except for YnfM, the scores of the hits in TCDB are very low, and annotations concerning substrate specificity are, consequently, highly hypothetical.

The gene *pagO,* coding for the Drug-Metabolite Transporter (DMT), was found to be active in *BAp* and *BSg*, pseudogenized in *BBp* and absent from *BCc*. Amino acids are the transported solutes of the PagO protein but this function remains very speculative given the very low match-score of the protein on the TCDB.

The Oxa1 protein (named YidC in *E. coli*), present in all four strains of *Buchnera,* is highly conserved and responsible for the membrane translocation of proteins (notably from the respiratory chain complex) in bacteria and mitochondria, associated or not with the secretion system. It does not seem to function as a transporter of metabolites [68].

With the exception of *BCc*, *Buchnera* possess a transporter for inorganic phosphate (encoded by the *pitA* gene).

*BAp* and *BSg* possess one MOP flippase, encoded by the gene *mviN,* for the export of several drugs and, possibly, N-metabolites and lipids, whereas *E. coli* possesses 8 of them. Besides mviN, *BBp* shows an additional MOP flippase, NorM. *BAp*, *BSg* and *BBp* possess the perM permease (autoinducer-2 exporter) that might be involved in the export of small solutes. These systems are totally absent in BCc.

Although no specific molecular function has been assigned to the corresponding orthologous genes in *E. coli*, the two genes *ychE* and *yhgN*, conserved in *BAp*, *BSg* and *BBp,* share sequence similarity with a putative neutral amino acid transporter and might be involved in the import of glycine, alanine, cysteine, threonine, serine, methionine and, possibly, asparagine as it has been experimentally detected in the hyperthermophilic archaeon *Thermococcus sp*. [69]. However, the hit-scores for these two proteins in the TCDB are low and, here again, specific solute transport annotation remains speculative.

No chloride specific transporter was annotated in *Buchnera,* whereas several have been found in *E. coli,* with the exception of a putative function of a similar domain within the YqhA protein (absent from *BCc*) encoded on the pLeu plasmid. More generally, no clearly defined anion permease was annotated, and anions were not found as cofactors of the *Buchnera* enzymes (see below).

#### Class 3. Primary active transporters

The ATP-Binding Cassette (ABC) superfamily of transporters include both uptake and efflux transport systems, all utilizing ATP-hydrolysis (without protein phosphorylation) to energize the transport. In *BAp* and *BSg*, four ABC transporters were found (together with two in *BBp* and one in *BCc*), whereas about 70 have been found in *E. coli*. The Znu system (import of Zn^2+^) is present in *BAp*, *BSg* and *BBp*. The MdlA/MdlB system is conserved in the four *Buchnera* strains and might be involved in the export of miscellaneous metabolites and lipids (multidrug-type). The Uup protein, only present in *BAp* and *BSg*, is an ATP-binding protein with an undetermined role, even for the orthologous protein in *E. coli*. The ATP-binding protein might complement the loss of such proteins in the other ABC systems in *Buchnera*, whereas a putative role in replication and transposon excision has been proposed in *E. coli* [70]. Lastly, the LolCDE system (export of lipoprotein) is present in *BAp* and *BSg,* although *lolE* (ATP binding protein) is found as a pseudogene in both lineages and LolA and LolB are absent. We can hypothesize that this incomplete system, lacking, among others, the solute-recognition protein, might have acquired a broader transport function in *Buchnera,* possibly with a lower efficiency.

The complete F-ATP synthase chain is found in *BAp*, *BSg* and *BBp* whereas it is absent from *BCc*. F-ATP synthase uses a proton gradient to drive ATP synthesis by allowing the passive flux of protons across the membrane, down their electrochemical gradient, and using the energy released by the transport reaction to synthesise ATP from ADP and inorganic phosphate.

The electron chain transporter, encoded by the *rnf* operon, is present in *BAp*, *BSg* and *BBp* but absent from *BCc*, highlighting that *BCc* is no longer able to oxidize and regenerate energetic compounds (ATP) from cellular respiration.

The four *Buchnera* strains each possess a complete or partial secretion system [71] involving 11, 11, 9 and 7 proteins for *BAp*, *BSg*, *BBp* and *BCc,* respectively. The complete *E. coli* secretion system comprises 11 genes. The secretion system is responsible for protein export across the inner membrane, from the cytosol to the periplasmic space.

The four *Buchnera* strains possess a T3-secretion system (flagellar apparatus, though lacking the flagellin core component) with 28, 28, 23 and 15 proteins in *BAp*, *BSg*, *BBp* and *BCc,* respectively.

#### Class 4 and 8. Group translocators and accessory factors involved in transport

*E. coli* possesses 29 different PTS transporters which aid in their survival and in the assimilation of a wide diversity of substrates. Apart from *BCc,* that totally lacks such systems, the three other *Buchnera* strains possess complete PTS systems only for the importation of glucose and mannitol, the two main carbon sources obtained from the cytoplasm of the bacteriocyte [37].

#### Class 9. Incompletely characterized transport systems and unreferenced proteins

The CorC cationic transporters (Mg^2+^, Co^2+^), encoded by the gene *ybeX,* are present in the four strains of *Buchnera.* Another gene, *yoaE,* was identified as homologous to *corC* in transportDB, but with a lower score. The role of YoaE as a cationic transporter remains highly hypothetical, but it is worth noting that it is one of the few small solute permeases conserved in all four *Buchnera* strains.

Four transmembrane proteins were also detected with no hit in the TCDB: HtpX, YqhA, YciC and YfgM, showing 4, 3, 6 and 1 transmembrane domains respectively. YfgM could be a periplasmic chaperone anchored to the outside of the inner-membrane and involved in the secretion of nascent proteins in the periplasmic space, associated with another chaperone, Pipd, found in *BAp*, *BSg* and *BBp* [72]. The three other proteins have orthologues in *E. coli* with uncharacterised functions, with the exception of YqhA for which a putative Cl^-^ domain has been assigned in EchoBase [73].

The heat-shock proteins, DnaK, and the GroESL heteromers were described as transporter facilitators by biasing the Brownian molecular agitation of the denatured proteins that cross the membrane through different channels [74]. A more specific role for channel-forming ions has also been described for some Hsc70 proteins [75]. These proteins are essential and are well conserved in all living organisms. DnaK and GroESL are highly expressed in the *Buchnera* cell [76], [77] and might have several functions linked with the symbiotic exchanges occurring between the two partners. However, no functional studies investigating their role in transport have, as yet, been carried out.

### Comparative genomics of transport capabilities across the range of bacterial diversity

**Figure 1:**
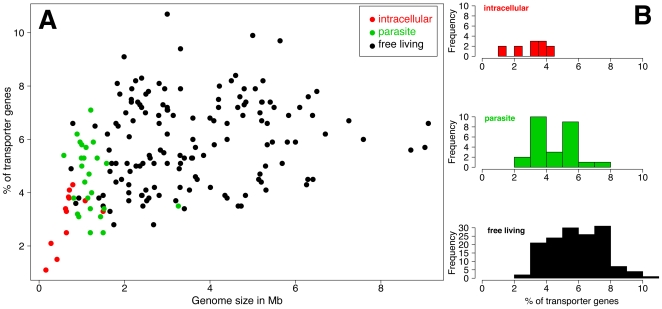
Comparative genomics of bacterial transporter genes. **A**: Plot of the percent of transporter genes versus genome size; **B**: distribution of the percent of transporter genes for 184 bacteria from the transportDB database (B). Red: intracellular obligate bacteria; Green: parasitic/pathogenic bacteria; Black: free-living bacteria.

Comparative genomics analysis of bacterial transport capabilities was performed using the “Compare Organisms” tool of the transportDB database [57]. The percentage of genes encoding transporters (among the whole list of CDS) was compared for 184 bacterial species spanning the range of bacterial diversity (Figure 1). The group of intracellular bacteria is characterized by a lower percentage of transporter genes, as compared with the group of parasitic or free living bacteria (Komolgorov distribution test, pvalue  =  0.001 and 10^−6^ respectively, Figure 1B) with percents ranging from 1 to 4 for the symbiotic group (n = 12), 2.5 to 7.1 for the parasitic group (n = 26) and, finally, 2.8 to 10.7 for the group of free-living bacteria (n = 146).

### Exploration of the metabolic network of *BAp* (input and output compounds) and the related transport capabilities in *Buchnera*

**Figure 2:**
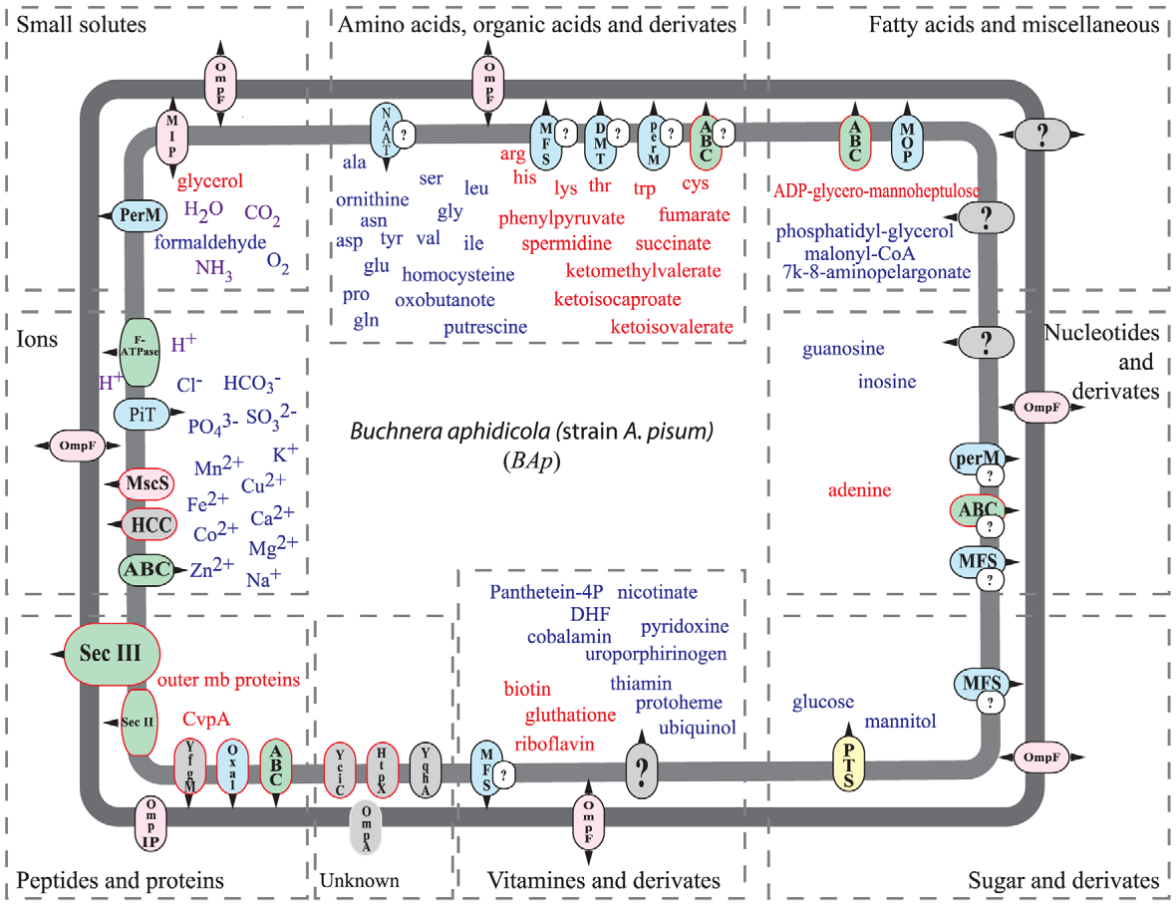
Schematics of transport capabilities in *BAp.* Input (blue) and output (red) compounds, predicted with the metabolic network analysis, are presented on the figure with their corresponding putative transporter families (dotted rectangles). Transporters are coloured according to their class: primary active transporters (green), secondary transporter (blue), group translocators (yellow), channels (pink), unknown (grey). Conserved transporters in the four *Buchnera* strains/species are outlined with red boxes. Small encircled question mark indicates that the corresponding transporter is an hypothetical candidate showing low sequence identity with the well annotated homologous reference in TCDB and/or for which the transported substrate was not known with a high accuracy for the orthologous reference sequence in TCDB (see table 1).

The metabolic network of *BAp* was systematically explored using the MetExplore information system, followed by manual refinements (see Materials and Methods section) to detect all the incoming compounds of the networks (inputs) and the most important outgoing ones (outputs). The complete results of this analysis are given in Tables S1. Table 2 summarizes the lists of the inorganic compounds (input and output), and Tables 3 and 4 present the input and output compounds for the other classes (amino acids, organic acids, nucleotides, sugars, vitamins, peptides and proteins, fatty acids and miscellaneous). These lists of inputs and outputs were compared with our transporter reannotation in *BAp* (table 1) and putative transporters were associated when possible to the different compounds in the last column of tables 2 to [Table pone-0029096-t003] 4. Figure 2 gives a schematic summary of the transport capabilities of *BAp*. Figure S1 summarizes the transport capabilities of *BSg*, *BBp* and *BCc* inferred from the transporter repertoire, presented in Table 1 for the three strains.

**Table 2:** List of the inorganic compounds and small solutes with their corresponding putative transporters in *Bap.*

Compound	Name	Super family of transporters (Protein name)[Import – Export]
***Ions***		
Anions	Cl, SO_3_, HCO_3_, PO_4_	MscS (YggB) [E], Porin (OmpF) [I-E], PiT (PitA) [I], *unknown (YqhA) [nd]*
Cations	H, Mg (37 a), Zn(34), Fe(28), Co(5), K(4), Ca(1), Cu(1), Na(1), Mn(9)	MscS (YggB) [E], Porin (OmpF) [I-E], ABC (ZnuC/B) [I], HCC (YbeX, *YoaE*) [E], F-ATPase (AtpA-H) [E]
***Small solutes***		
CO_2_	Carbon dioxide	Diffusion
O_2_	Oxygen	Diffusion
H_2_O	Water	Diffusion, MIP (GlpF) [I-E], perM (YdiK) [E], porin (OmpF) [I-E]
NH_3_	Ammonia	
glycerol	glycerol	
CH_2_O	formaldehyde	

a : number of enzymes in *BAp* using the cation as a cofactor (from HAMAP project, http://www.expasy.org/sprot/hamap/). Transporters written in italics are hypothetical candidates showing low sequence identity with the well annotated homologous reference in TCDB and/or for which the transported substrate was not known with a high accuracy for the orthologous reference sequence in TCDB (see table 1).

**Table 3 pone-0029096-t003:** Summary of the input compounds, found with MetExplore, from the analysis of the metabolic network of *BAp* and determination of the corresponding putative transporters.

Input compounds	Main biosynthesis pathways in *Buchnera*	Putative importers
***Amino acids, organic acids and derivates***		
Ala	proteins	*NAAT (YchE, YhgN) [I], unknown (YqhA) [nd]*, Porin (OmpF) [I-E]
Asp/Asn	Thr, Lys, Arg, proteins	
Glu/Gln	Ser, Lys, Trp, Phe, His, Arg, gluthatione, proteins	
homocysteine	Met, SAM/SAH	
Pro	proteins	
Ser/Gly	Cys, Trp, gluthatione, proteins	
Tyr	proteins	
Val/Leu/Ile	proteins	
2-oxobutanoate	Ile	
putrescine	purine/pyrimidine	
***Nucleotides and derivates***		
guanosine	purine/pyrimidine	Unknown (inner mb), Porin (OmpF) [I-E]
inosine	purine/pyrimidine	
***Sugar and derivates***		
glucose	Val, Leu, Trp, Phe, His, purine, FAD and derivates	PTS (PtsGI/H, Crr), Porin (OmpF) [I-E]
mannitol	idem as glucose	
***Vitamins and derivates***		
cobalamin	Met	unknown (inner mb), Porin (OmpF) [I-E]
DHF (or 7-8, dihydropteroate)	Met, purine/pyrimidine and general methyl donor/acceptor	
nicotinate	NAD general proton acceptor	
pantetheine-4P (or CoA)	general cofactor	
pyridoxine	general enzymatic cofactor	
thiamine	sulfur relay transfer reaction	
ubiquinol	aerobic respiration	
uroporphyrinogen and protoheme	cytC for respiration and detoxification	
***Peptides, proteins, fatty acids and miscellaneous substances***		
7-keto-8-aminopelargonate/malonyl-CoA	biotin	unknown (inner mb), Porin (OmpF) [I-E]
L1-phosphatidyl-glycerol	cardiolipin	Unknown (inner and outer mb)

More details are given in Tables S1.

Transporters written in italics are hypothetical candidates showing low sequence identity with the well annotated homologous reference in TCDB and/or for which the transported substrate was not known with a high accuracy for the orthologous reference sequence in TCDB (see table 1).

**Table 4:** Summary of the output compounds, found with MetExplore, from the analysis of the metabolic network of *BAp* and determination of their putative transporters.

Output compounds	Main biosynthesis pathways in the aphid	Putative exporters
***Amino acids, organic acids and derivates***		
2-keto-3-methyl-valerate	Ile, proteins	*MFS (YbaI, YaJR, TsgA, YnfM) [E], DMT (PagO) [E], perM (YdiK) [E], ABC (MdlA/B) [E]*, Porin (OmpF) [I-E]
2-ketoisovalerate	Val, proteins	
2-ketoisocaproate	Leu, proteins	
Arg	proteins	
Cys	Homocysteine, Met, proteins	
His	proteins	
Lys	protein	
phenylpyruvate	Phe	
Thr	Ile, proteins	
Trp	proteins	
spermidine	purine/pyrimidine	
Fumarate and succinate	central metabolism (elimination is needed as the TCA cycle is incomplete in *Buchnera*)	
***Nucleotides and derivates***		
adenine	purine/pyrimidine	*MFS (YbaI, YaJR, TsgA, YnfM) [E], perM (YdiK) [E], ABC (MdlA/B) [E]*, Porin (OmpF) [I-E]
***Vitamins and derivates***		
biotin	general cofactor	*MFS (YbaI, YaJR, TsgA, YnfM) [E], perM (YdiK) [E], ABC (MdlA/B) [E]*, Porin (OmpF) [I-E]
riboflavin	general proton acceptors (FAD, FMN)	
***Peptides, proteins, fatty acids and miscellaneous substances***		
ADP-glycero-mannoheptulose	putative shuttle in the LPS biosynthesis	ABC (MdlA/B) [E], MOP (MviN) [E]
Outer membrane proteins	outer membrane transport systems	Sec [E], T3 [E], Porin (YaeT) [E], Oxa1 IMP (OxaA) [E], ABC (LolD/C) [E], YfgM [E]

More details are given in Tables S1.

Transporters written in italics are hypothetical candidates showing low sequence identity with the well annotated homologous reference in TCDB and/or for which the transported substrate was not known with a high accuracy for the orthologous reference sequence in TCDB (see table 1).

#### Transporters for ions, inorganic and small solutes

Anion and cation transporters are essential for maintaining cell osmotic pressure, electrochemical membrane potential and pools of cationic enzyme cofactors, as well as for metabolism (notably for sulfate and phosphate). A systematic research, using specific cofactor queries on the SwissProt HAMAP database, has indicated the requirement, as a cofactor, of the following cations: Mg, Zn, Fe, Co, K, Ca, Cu, Na and Mn, for at least one of the enzymes of *BAp* (Table 2). Generic ion permeases and porins are present in *BAp,* and in the three other *Buchnera* (MscS and OmpF); however, the considerable diversity of specific ion transporters found in *E. coli* seems to be lost in *BAp,* which has conserved only specific Zn, Mg/Co, and inorganic phosphate transporters. It is important to note that no specific Cl^-^ channel was identified in *BAp*, although a putative domain is described in the YqhA protein. Moreover, *BAp* seems to have lost the capability to build electrochemical gradients across membranes (H^+^, Na^+^, K^+^) as no primary pump is encoded in the genome. All secondary transporters (class 2 in Table 1), therefore, need an ion gradient primarily energized by a supposed pH gradient that is probably maintained here by the host (see below the section on intracellular pH).

*BAp,* and the three other *Buchnera* strains, lack a specific ammonia transporter (encoded by *amtB* in *E. coli*) even though ammonia is probably an important compound produced by the aphid that is recycled within the amino acid biosynthesis pathways [78], [79], [80]. It is not clear whether ammonia circulates between the two partners and in which direction, as most of the ammonium recycling, due to the coordination of glutamine synthase and glutamate synthase, occurs in the bacteriocyte [79]. The absence of an ammonium transporter is not specific to *Buchnera*, as the other small-genome symbionts also lack this kind of transporter. Ammonia, as well as water, glycerol and formaldehyde, might diffuse passively across the *Buchnera* membrane or it might be transported through the general porins (MIP, PerM and OmpF).

#### Transporters for amino acids, organic acids and derivates

Amino acid exchange is the keystone of the symbiotic relationship between aphids and *Buchnera*. The biosynthesis pathways for non-essential amino acids have been lost in *Buchnera* and so they must be imported into the cell. The essential amino acids are produced in the *BAp* cell almost exclusively from aspartate or glutamate and glucose/pyruvate but, nevertheless, some pathways are incomplete and apparently there is a need to shuttle precursors to the host cell to achieve complete biosynthesis [36]. Surprisingly, the capabilities of amino acid transport in *BAp* seem to be very limited. No specific amino acid transporter was clearly found, although putative neutral amino acid importers encoded by *ychE* or *yhgN* were found with a low hit-score on TCDB. Hence, the means of amino acid importation remains largely unclear in *BAp* and the transport of all amino acids seems to rely on general transporters, such as the OmpF porin in the outer membrane, and possibly some MFS, DMT, PerM and ABC exporters across the inner membrane.

#### Transporters of nucleotides and their derivates

The biosynthesis pathway of nucleotides is partial, in *BAp,* in that it needs to import guanosine and inosine, whereas it produces adenine for the host, as suggested by the genomic analysis of *A. pisum* by Ramsey *et al*. [81]. As for the amino acids, the only corresponding transporter in the outer membrane is the general porin OmpF. It can be hypothesized that some general exporters (PerM, ABC and MFS) might export adenine; however, the import of guanosine and inosine across the inner membrane in *BAp* remains unclear.

#### Transporters of sugars and their derivates

Sugar transport is complete in *BAp* due to the combination of the general porin (OmpF) with the functional PTS systems, for glucose and mannitol import across the outer and inner membranes, respectively.

#### Transporters of vitamins and their derivates

The riboflavin (vitamin B2) biosynthesis pathway is complete, in *BAp,* for GTP and ribulose 5-P. Our hypothesis is that *BAp* synthesizes riboflavin for *A. pisum,* although specific exporters have not been detected. MFS (YabI) is one putative candidate for the export of this vitamin across the inner membrane.

The biotin (vitamin B7) biosynthesis pathway has been recently elucidated [82], beginning with malonyl-CoA through to the fatty acid elongation pathway. *BAp* partially conserved the pathway, encoding *fabI*, *fabB*, *fabG*, *bioA*, *bioD* and *bioB* genes but lacking *fabH*, *fabZ*, *bioH*, *bioF* and *bioC*. The pathway might be complete in *BAp* supposing that paralogous enzymes, or enzymes that have lost some substrate specificity, might catalyse the missing reactions; however, this question, including fatty acid and membrane biogenesis, has not yet been properly investigated in *BAp*. It is important to note that the pathway is almost complete in *BBp,* lacking only the *fabH* gene. Hence, biotin precursors in *BAp* might be either malonyl-CoA (if the pathway is complete) or 7-keto-8-aminopelargonate (if only the end of the pathway is functional). However, the latter precursor does not seem to be produced by aphid metabolism, as analysed through the AcypiCyc database.

Haem biosynthesis in *BAp* is mostly degraded. However, the *BAp* genome encodes the four subunits for the cytochrome o complex, as well as protohaem farnesyltransferase (*cyoE*), the enzyme that places a farnesylethyl group on protohaem to form the cytochrome o prosthetic group. In addition, the genome encodes sulfite reductase, which has sirohaem as the prosthetic group, together with the enzymes that synthesize sirohaem from uroporphyrinogen III (*cysG*) [83]. From these observations, it can be inferred that two intermediate precursors must be imported from the *A. pisum* bacteriocyte into the bacterial compartment: the uroporphyrinogen III and the protohaem. The question of the import of these compounds across the inner membrane is still open as no corresponding importers were found in the *BAp* genome.

The nicotinate (vitamin B3), panthotenate (vitamin B5) and folate (vitamin B9) pathways are also incomplete, in *BAp,* hence the necessary import of nicotinate for the biosynthesis of NAD and NADP, pantheteine-4P for Coenzyme-A and dihydropteroate for DHF and THF, respectively. Interestingly, the genes coding for the pathway from nicotinate to NAD and NADP have been conserved, whereas those coding the pathway from nicotinamide have been lost. Thus, we can hypothesize that the imported precursor of NAD, and its derivatives, in *BAp* is probably nicotinate, rather than nicotinamide

Biosynthesis pathways of thiamine (vitamin B1), pyridoxine (vitamin B6) and cobalamin (vitamin B12), as well as those of ubiquinol and ubiquinone, are absent in *BAp* so the direct import of these compounds is essential as they are readily used by encoded enzymes, as verified through HAMAP/UniProt annotation (see Tables S1). Specific transporters for these compounds have been found in *E. coli* but not in *Buchnera*.

Nicotinate, panthotenate and folate, as well as thiamine, pyridoxine and cobalamin, are probably found in the phloem sap by the host, and they have all been shown to occur in the phloem sap exudates of some plants [84], [85]. Thus, they would normally be made available to *BAp*, although specific inner-membrane importers have not been identified in the genome of the bacterium.

#### Transporters for peptides, proteins, fatty acids and miscellaneous substances

The secretion systems (inner-membrane exporters of proteins) and T3 (flagellum) are almost complete in *BAp.* Four additional putative protein transporters were also found in this genome, one ABC transporter for lipoproteins (encoded by lolDC), the Oxa1 protein and the chaperone YfgM, all within the inner membrane, as well as the YaeT protein within the outer membrane. It is important to note the absence in *BAp* (and in the other *Buchnera* strains), as compared to *E. coli,* of the protein assemblers, LptE and LptD, for the lipopolysaccharide (LPS) component of the bacterial envelope, several chaperone proteins associated with lipoproteins (Lpp, Skp), and the sigma-E factor controlling protein processing in the extracytoplasmic compartment (encoded by *rpoE*).

Lipid biosynthesis in *BAp* is very poorly conserved. The LPS biosynthetic pathway is incomplete, producing lipid-A precursors such as ADP-D-glycero-D-manno-heptulose. This compound might be exported across the inner membrane using the MOP flippase MviN or the MdlA/B ABC transporters. Cardiolipin is produced by cardiolipin synthase (encoded by the *cls* gene) from L1-phosphatidyl-glycerol. However, the import of such a compound is still not clear.

### Structural analysis of the bacterial and symbiosomal membranes

#### Comparative membrane topology of symbiosomal vesicles of three aphid species

**Figure 3:**
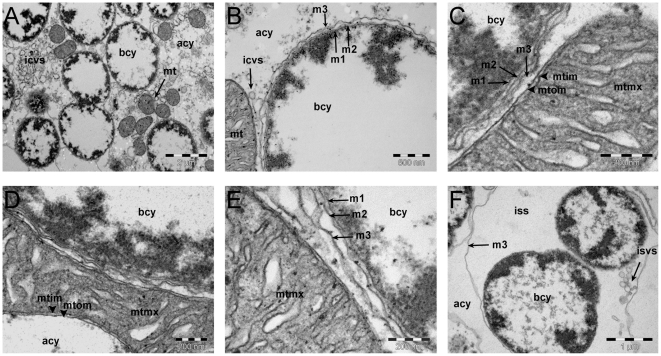
Structural analysis of bacterial (*Buchnera*) and symbiosomal membranes in the pea aphid. A: low magnification view of a bacteriocyte cell showing *Buchnera* cells, numerous mitochondria and the vesicular system within host-cell cytoplasm; B: enlarged view of *Buchnera* and mitochondria, showing the three membranes of the *Buchnera* symbiosome; C: higher magnification view of membrane organization, with the inner/outer double membrane of a mitochondrion (with matrix and cristae), and the triple membrane surrounding *Buchnera*; D: another view of *Buchnera* and adjacent mitochondrion multiple membranes; E: highest magnification view of membrane organization, showing the m1/m2/m3 triple membranes delineating *Buchnera*. All intermembrane distances (see Results) were measured at the scales indicated with Olympus Analysis® software; F: lower magnification view of older maternal bacteriocytes. In all figures, the following abbreviations are used: **bcy**: *Buchnera* cytoplasm, **acy**: aphid bacteriocyte cytoplasm, **mt**: mitochondria, **mtmx**: mitochondrial matrix, **mtim**: mitochondrial inner membrane, **mtom**: mitochondrial outer membrane, **m1**: *Buchnera* inner membrane, **m2**: *Buchnera* outer membrane, **m3**: symbiosomal membrane surrounding the *Buchnera* cells, **Nu**: nucleus, **ssm**: symbiosome (symbiosomal vesicle), **iss**: intrasymbiosomal space (extracellular to *Buchnera*), **isvs**: intrasymbiosomal vesicle, **icvs**: intracytoplasmic vesicle.

**Figure 4:**
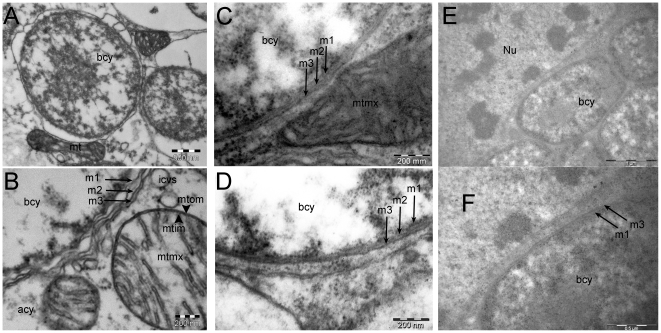
Comparative analysis of symbiosomal membranes in aphid bacteriocytes from *Acyrthosiphon pisum* (A, B), *Cinara cedri* (C, D) and *Baizongia pistaciae* (E, F). The canonical three membranes, shown in Figure1, are visible in *A. pisum* and *C. cedri* (B, D arrows) and should be compared with the mitochondrial two-membraned envelope (B, arrowheads). In *Baizongia pistaciae*, no three-layer system was identified. From this, and the transporter set data, homology can be proposed with the m1/m3 (inner membrane/symbiosomal membrane) of the other aphid species (arrows in F); abbreviations are as in Figure 1.

Notable differences in the transporter gene content between the four *Buchnera* strains analysed in this work led us to make a structural analysis of the symbiosomal membrane systems in *BAp*, *BCc* and *BBp* (*BSg* being almost similar to *BAp,* and phylogenetically very close, was not included in the analysis). The canonical three-membrane system, already described for aphid primary endosymbionts, is presented for *BAp* in Figure 3A-C. Although they differ, mostly in the type and the total number of their transporter sets, *BAp* and *BCc* undoubtedly display the canonical three-membraned system previously described for aphid primary symbionts (Figure 4A-D). However, despite careful examination, we were not able to detect any three-layer organization in *BBp* (Figure 4E-F). As resolution was lower for *BBp* and *BCc* transmission electron microscopy (TEM) images, several preparations were analysed and an automated procedure of image filtering and membrane detection was applied on 9 images (6 for *BBp* and 3 for *BCc*) giving similar representations and coherent intermembrane distances with two- and three-layer organisation in *BBp* and *BCc*, respectively (Figure S2).

#### Membrane topology within the *Buchnera*/symbiosomal membrane boundaries

Trying to build a general model of molecular transport within the bacteriocytes led us to the question of whether the three-membraned system, characterizing *Buchnera*'s inclusion within the symbiosomal vesicle, could give rise to transport-associated “contact points” similar to those described in mitochondrial membranes (an inner/outer two-membrane system). Extensive examination of the membrane system surrounding *Buchnera,* in young adult maternal bacteriocytes, did not identify any of the numerous potential contact points, and inner-symbiosomal transmembrane distance measurements at these narrow parts (Figure 3 C–E) revealed means of 37.3 nm ± 7.2 in *BAp* (41.4±6.2 in *BCc*). These are probably not compatible with the building of complex integrated transport systems, such as have been extensively studied in mitochondrial membranes (measured at 14.8 nm±2.8 in mitochondria of *A. pisum*, Figure 3 C–E), corresponding to the TIM/TOM or ANT supra-molecular complexes [45]. Figure 3F presents older maternal bacteriocytes with multiple bacteria within a single loose symbiosomal vesicle delineated by its m3 membrane. Intrasymbiosomal vesicles, as seen here, are frequent in such symbiosomes often containing dividing *Buchnera* and they are suggestive of an increased metabolic activity in this compartment that might be linked with the end of symbiosis in old aphids, as previously suggested by Nishikori *et al*. [86] in old alate aphids.

#### pH gradient within bacteriocytes

**Figure 5:**
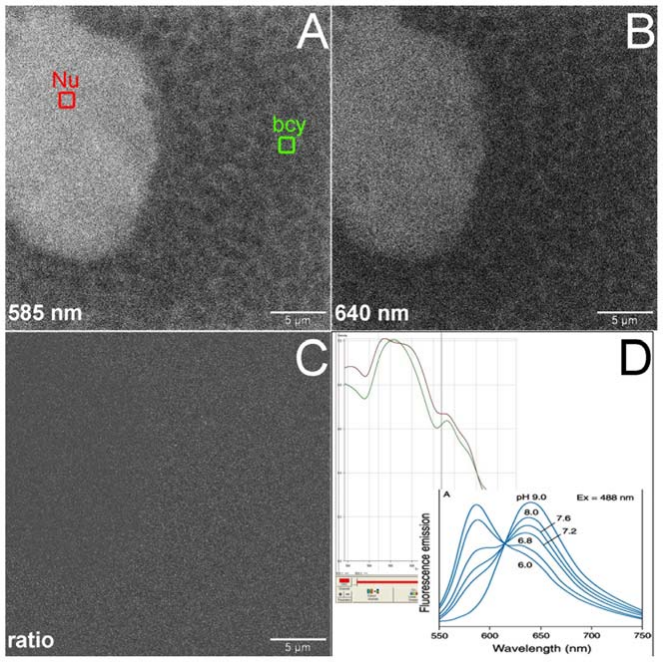
Confocal microscopic analysis of pH fields inside *A.\ pisum* maternal bacteriocytes. Analysis of pH gradients within live bacteriocytes incubated with the SNARF®-AM ester pH sensitive fluorescent probe (see Methods). The main objective was to detect any pH differences between *Buchnera* cytoplasm (bcy, green) and aphid cytoplasm (acy) surrounding the *Buchnera* symbiosomes or nuclear (Nu, red) fields. **A**: image of the 585nm emission window (SNARF® maximum emission at acidic pH) showing the outline of packed *Buchnera* symbiosomes filling the cytoplasm of the bacteriocyte. The brighter object is the bacteriocyte nucleus; **B**: image of the 640nm emission window (SNARF® maximum emission at alkaline pH); **C**: ratiometric analysis of the same image at 585/640 ratio, showing uniform density, hence the absence of gradients between the *acy* and *bcy* areas; **D**: emission spectra of SNARF® probe controlled, *in situ,* in the *bcy* (red) and *Nu* (green) fields shown in A (left inlet) and the control emission spectra of the SNARF® probe (right inlet).

The analysis of intracellular pH potential within the *A. pisum* bacteriocyte, through labelling with the SNARF®-1 pH-sensitive fluorescent probe, revealed that no differential pH fields could be detected between the host cell cytoplasm and the bacterium cytoplasm (Figure 5A–C). The emission spectrum of the SNARF® probe was checked, *in situ,* within both cytoplasmic fields to ensure that the probe had penetrated all the compartments (Figure 5D inlets, SNARF® emission spectra in aphid cell and *Buchnera* cell cytoplasms). Finally, the ratiometric analysis of confocal images (ratio of 585/640 nm emissions), that should detect the bacteria within the bacteriocytes if the pH of their cytoplasm was significantly shifted, did not reveal any contrast (Figure 5C). However, the resolution of confocal analysis did not allow us to detect pH shifts across the symbiosomal extrabacterial compartment, which may constitute the active compartment where H^+^ ions could be pumped by the host fuelling, in turn, H^+^ secondary transporters or ATP synthesis.

#### Ultrastructural immunogold detection of GroEL within adult bacteriocytes

Our microscopic observations clearly indicate that, out of the three potential locations within the bacteriocytes (intracytoplasmic *Buchnera*, membrane-associated *Buchnera* and intracytoplasmic aphid host cell), GroEL gold label was almost always associated with *Buchnera*'s intracytoplasmic electron-dense material (Figure S3, C–F). This observation confirms that the activity of this protein is confined to the *Buchnera* cytoplasm, compatible with its function as a chaperone rather than with a function related to transport across membranes. While only marginal gold staining was found in the bacteriocyte cytoplasm (Figure S3, C or E), some remnant multi-vesicular bodies, representing decaying *Buchnera,* were found with extensive electron-dense associated labelling (Figure S3, F). This material could ultimately constitute the source of released GroEL, although no detection of the label was observed either in the bacteriocyte's vesicular system, or in the surrounding sheath-cells (data not shown).

## Discussion

The study of the genome sequence of *Buchnera,* belonging to different aphid lineages, and the comparison with its free-living relatives, such as *E. coli*, has allowed us to carry out a comprehensive analysis of the retained transport system in these endosymbionts, which have undergone massive gene decay during their adaptation to intracellular life. Indeed, transport is one of the most important functions in this symbiosis, where the main role of the bacteria is the provision of nutrients for their hosts whilst importing, from the aphid, the metabolites they cannot synthesize. In addition, the structural membrane organization of different *Buchnera* has been analyzed and compared for the first time.

Although our metabolic network analysis revealed high complementarity between the aphid hosts and their associated bacteria (*i.e*., several pathways are often shared by the two symbiotic partners, with exchanges of compounds between them), the transport function in *Buchnera* is based on a very low diversity of transporters, as compared to free-living bacteria. The number of transporter systems varies between 12 and 34 in *Buchnera* whereas about twenty times more have been described in *Bacillus subtilis* and *E. coli* [57].

This observation is true for all four *Buchnera* strains that we have analysed in our comparative study: these intracellular bacteria show a lower diversity of transporters (estimated roughly from the percentage of transporter genes in the genome) when compared with parasitic and free-living bacteria, and this is probably not simply due to the drastic genome reduction that has occurred in all endosymbionts. Indeed, the ratio of transporter genes is much more closely correlated with the life style of the bacteria (Figure 1, R^2^ = 0.22, pvalue  =  10^−10^) than with the genome size; the correlation between transporter gene count and genome size is very low in free-living bacteria, despite a large range of genome size variation within the group (Figure 1, R^2^ = 0.03, pvalue  =  0.03).

It is important to note that the true number of transporters is probably underestimated in the transportDB database. As an example, the percentage of transporter genes in *BAp* is 3.4% in transportDB, whereas we have estimated it to be 15%, after manual refinement in this work (see Results section). As this bias might not be constant in all phylogenetic groups of bacteria, we restricted the analysis to the group of gamma-proteobacteria in transportDB and, consistently, the intracellular symbiotic bacteria revealed about half the number of transporter genes per Mb of genome when compared to the group of free-living bacteria (data not shown).

The low diversity of transporters in *Buchnera* (and more generally in symbiotic bacteria, as shown in our comparative analysis) is a result that might seem counterintuitive, as the intracellular symbiotic equilibriums are based on host-symbiont exchanges. However, this is also an indication, on the one hand, that the genetic diversity of the transport function in bacteria is probably highly specialised to respond to stimuli from the external environment (resistance, pathogenicity, sensors, detoxification etc.) whilst a “housekeeping” metabolism probably needs a less diversified transporter set. On the other hand, the extreme reduction of transporter diversity in *Buchnera* (as in the other intracellular symbiotic bacteria) can also be explained by the relaxed selective constraints occurring in the context of intracellular symbiosis. In addition to the simplification of their metabolic network, transported solute diversity might also be reduced, as compared to that of extracellular bacteria.

Paulsen et al. [87] revealed that, in prokaryotes, transporters represent about 10% of the encoded CDS, with global variations according to the primary forms of energy generated via the metabolic pathways (bioenergetics of transport), ecological niches and substrate availability. In another study, the same group of researchers stated that the organisms with the highest percentages of primary transporters (class 3 in Table 1) are those that generate energy by substrate-level phosphorylation and lack electron transport and a Krebs cycle, such as *Mycoplasma*, *Borrelia* and *Treponema*. These organisms generate ATP as their primary source of energy and generate a proton motive force and ion gradients (*e.g*., H^+^ and Na^+^ gradients) secondarily, using ATP hydrolysis. Such energy is most frequently used to drive nutrient uptake and maintain ionic homeostasis [88].

In *Buchnera*, the highest diversity of transporters, although quite poor, is found in the group of secondary transporters using ion-gradient forces with about 13 systems, described and linked with all categories of metabolites (class 2 in Table 1, blue in Figure 2). *Buchnera* strains have lost the capability to build their ion gradients. It is probable that these bacteria use ion gradients generated by the host, as suggested by Paulsen *et al*. [88] for *Rickettsia*, *Chlamydia* and *Treponema*, (this is the case for F-ATP synthase, using a proton gradient). It is striking that *BCc*, which has lost the F-ATP synthase complex and, possibly, the proton impermeability of its inner membrane, has consequently lost all the genes encoding secondary transporters.

In *Buchnera*, the class of primary transporters consuming ATP (class 3 in Table 1, green in Figure 2) comprises 4 ABC transport systems for ions (putatively for divalent cations, such as Zn^2+^ and its analogues) and for lipids and lipoproteins, whereas about 70 are found in *E. coli*. It is noteable, again, that *BCc*, having lost the capacity to regenerate ATP from ADP, has consequently lost 3 out of the 4 ABC transport systems (except *mdlA*/*mdlB*). In this class of active transporters, the secretion system is responsible for the translocation of membrane-bound proteins across the inner membrane. The secretion system is conserved (although reduced to a minimal composition) in the four *Buchnera* strains, as is the T3-secretion system (flagellum lacking its *fliCD*-encoded filament). Toft and Fares [89] revealed that, within the flagellar assembly pathway, only proteins involved in protein export (T3-secretion system and the basal body) have been maintained in the majority of endosymbionts. Based on molecular evolution analyses, they suggested that the genes of flagellum have diverged functionally in order to specialize in the export of proteins from the bacterium to its host. Indeed, it has been shown that ΔfliC mutants of *E. coli* were able to export recombinant protein [90]. Also, flagellar genes are highly expressed in *BAp* [91] and flagellum systems were shown to be abundant at the periphery of *Buchnera* cells [71], a high expression that was also confirmed in the recent, and first, proteomic analysis of the aphid bacteriocyte (Poliakov et al 2011). However, the transport/export function of this system has not yet been functionally demonstrated in *Buchnera*.

The channel class (class 1 in Table 1, pink in Figure 2) comprises general transporters for ions (MscC), small solutes (MIP) and the OmpF porin. These channels are probably keystones of the transport function in *Buchnera*, as the very general Omp porins are the only outer-membrane transporters of *Buchnera*. It is important to note that at least one member of the outer membrane OmpA-OmpF porin (OOP) family is conserved in all gram-negative bacteria analysed in TransportDB, except in *BBp*. Interestingly, Poliakov et al [92] determined that OmpF is one of the most abundant proteins in *BAp* and it is ranked sixth out of the 400 proteins identified in this proteomic analysis.

The group translocator class (class 4 in Table 1, yellow in Figure 2) comprises only the glucose/mannitol PTS transport system in *BAp*, *BSg* and *BBp*. In *BCc*, this transporter system is absent, and a relatively passive diffusion of sugars across the inner membrane has been invoked by Perez-Brocal *et al*. [11] as a probable solution. This situation is most intriguing and would normally hamper the core function of *Buchnera*, which uses sugar carbons to build up essential amino-acid backbones for its host [37]. An alternative route could be an extensive importation of pyruvate from the host and/or the use of the hitherto uncharacterized conserved transporter for carbon source import (YciC).

Because of the highly reduced genome of *BCc,* the complete repertoire of genes involved in the transport function reveals only a small number which have been conserved in the four *Buchnera* strains (red outline boxes in Figure 2), most of them belonging to the protein-processing class. Outside this category, only four genes are involved, namely the ion permeases *YggB* (MscS family) and *YoaE* (HCC family), the ABC cassette *mdlAB* and the unknown *yciC* gene, which represent good candidates for future functional studies due to the fact that this core function is still uncharacterized in minimal living cells. It is of note that the two unknown transporters, YfgM and YqhA, were found in the four *Buchnera* strains (except for the absence of YqhA in *Bcc*). These two genes were found to be highly differentially expressed in *BAp* in experiments comparing aphids reared on diets with various amino acid concentrations [93], [94]. However, their function in amino acid transport is probably indirect as YfgM is associated with protein secretion and YqhA does not show any similarity with other previously identified amino acid or organic acid transporters.

The structural analysis performed in this work confirmed, for *BAp* and *BCc,* the existence of a three-membraned system for the symbiotic compartment within the bacteriocyte (*i.e*., the two bacterial membranes surrounded by the host symbiosomal membrane). However, TEM observations did not support the existence of contact points, which would allow the coupling of the transport across the host and bacterial compartments, as occurs in mitochondria. Furthermore, *Buchnera* does function more like a standard intracellular bacterium (cytoplasm at pH 7) rather than like an integrated organelle, with an intramatricial alkalinisation energizing the specialised transmembrane transport of ions or metabolites, as is the case in mitochondria and chloroplasts [95].

Finally, we investigated the potential role of the GroEL protein in the transport function of *Buchnera*. However, our microscopic observations clearly indicated that, out of three potential locations within the bacteriocytes (intra-cytoplasmic *Buchnera*, membrane-associated *Buchnera,* and intracytoplasmic aphid host-cell), GroEL gold-label was almost always associated with *Buchnera*'s intracytoplasmic electron-dense material (Figure S3, C–F). This subcellular localization suggests more a chaperone function for GroEL rather than an active involvement as an export-related protein. Moreover, GroEL does not seem to be massively exported or related to a translocation-related function, and its detection in the aphid body or haemolymph may be a result of bacterial cell turnover. These observations are consistent with the recent work of Bouvaine et al. [96] showing, in *BAp* and *Buchnera* from *Rhopalosiphum padi,* that GroEL is almost absent from the heamolymph and the digestive tract and unlikely to contribute to virus transmission by aphids. The identification of glycosylated GroEL [49], potentially marking the transit of the GroEL polypeptide through a eukaryotic endomembrane/Golgi compartment, is still an unsolved mystery.

The two aphids, *A. pisum* and *S. graminum,* are characterised by a high adaptive potential, being oligophagous and living on herbaceous plants. Consequently, their *Buchnera* strains conserve the richer pattern of transporter diversity, as compared to the two other strains. The most striking observation in *BAp* and *BSg* is the absence of identified importers for the main “symbiotic” compounds (*i.e*., amino acids, shuttle organic acids and vitamins). In comparison, *E. coli* possesses about 11 different channels, 70 MFS and about 42 specific transporters for amino acids. Hence, we can speculate that the molecular evolution of transporters might have increased solute pattern non-specificity of the remaining transporters, and probably decreased their efficiencies. Another hypothesis is the complementation of the transport deficiency by transporter systems encoded by the host and addressed/targeted to the bacterial membranes. Two aphid transporter proteins were indeed enriched in fractions of purified *Buchnera* cells, as recently described by Poliakov et al. [92]: ACYPI001015 (a mitochondrial α-ketoglutarate/malate carrier protein, misspelled as ACYPI001025) and ACYPI002559 (a MFS-type monocarboxylate transporter). The results found in our work demonstrate a crucial lack of such a transporter(s) in the inner membrane of *Buchnera* (*e.g*., for the transport of glutamate, aspartate, succinate and fumarate). The involvement of aphid proteins in the transport/membrane system of *Buchnera* remains very speculative, and Poliakov *et al.* [92] did not find any significant trace of such a localisation, nor even any strong evidence of individual enrichments, when analysing the variation coefficients of the cited candidate proteins across their biological replicates.

*B. pistaciae* is a more specialised aphid, with an ecologically predominant phase of gall-formation and, thus, a source of phloem modification on its host tree [18]. Its symbiotic bacterium, *BBp,* is also very specific since its symbiosomal structure seems organized only as a double membrane system (Figure 4E, F and Figure S2). As genomic analysis reveals the absence of all the outer-membrane components found in the other *Buchnera* strains (Table 1 and the Bam family, as well as the two non-transport associated components, *smpA* and *surA*), we hypothesized here the loss of the outer bacterial membrane in *BBp*. On the other hand, *BBp* is the only bacterium among those we analysed to have the Pal protein encoded by gene *BBp_282* (not conserved in *BAp*, *BSg* and *BCc*), probably involved in the anchoring of the peptidoglycan to the bacterial membrane. Pal is usually attached to the outer-membrane [97]. However the Pal sequence in *BBp*, including the peptide signal anchoring the protein to the membrane by a conserved lipoylated cysteine residue, has strongly diverged compared to that of *E. coli* (data not shown). Its localisation, and its role, remain unknown in *BBp* but its features may indicate neo-functionalization. Compared with *BAp* and *BSg*, *BBp* has also lost the MIP aquaporin, the DMT transporter and two of the ABC transport systems, probably correlated with the simplification of its metabolic network due to the specialisation of its aphid host as a gall-feeding species (this point has not been analysed precisely in this work). The loss of several genes within the secretion system and the flagellum might be related to the double membrane reorganisation of the bacterium. It is worth noting that CvpA (channel-forming colicin V), a putative transporter system for the colicin V toxin, is integrally conserved in *BBp*, whereas it is truncated in *BAp* and *BSg* (and absent in *BCc*). Here again, neo-functionalization could be suspected in this group of conserved proteobacterial membrane proteins, and this may have occurred in independent symbiotic taxa as a complete *cvpA* is present in the tse-tse fly primary symbiont *Wigglesworthia* [56].

Finally, *BCc* is probably the most specialised and astonishing bacterium among the four *Buchnera* strains analysed in this work. Its very reduced gene repertoire is correlated with the drastic reduction in the number of transport systems, despite the persistence of a three-membrane organisation (although no gene devoted to membrane formation has been retained in BCc). The genome reduction in this bacterium is probably explained, on the one hand, by the specialised biology of the host aphid, being strictly subservient to its cedar host-plant [98] and, on the other hand, by the persistence of the obligate secondary endosymbiont “*Ca.* Serratia symbiotica” that complements the primary endosymbiont's deficiencies. Such a model of double obligate endosymbionts, resulting in the simplification of the bacterial metabolic networks, was analysed systemically in the sharpshooter *Homalodisca coagulata* [99]. Hence, *BCc* has lost all its secondary transporters (because the ion gradients are probably not maintained in the bacteria) and most of its primary transporters (the bacteria being totally dependent on the regeneration of ATP from ADP). The secretion system and T3 are both conserved, though severely reduced. LspA (the lipoprotein signal peptidase), SurA (the chaperone involved in the correct folding and assembly of outer membrane proteins) and degP (the serine endopeptidase for outer membrane proteins) are absent in *BCc,* although present in *BAp* and *BSg,* indicating probable deficiencies in the assembly of the outer membranes in *BCc*. Similarly, the phosphorylation-dependent PTS systems for sugar import have been lost. The loss of ATP synthase, the respiratory chain and the PiT transporter has major consequences regarding the dependency of *BCc* on its host cell, even to maintain its oxidative level (ATP, NADH, FADH). Perez-Brocal *et al.* [11] mentioned that *BCc* might be close to being a a free-diffusing cell (corresponding with the loss of OmpF, as shown here), in which most metabolites can passively exchange through a highly simplified envelope. Another possibility would be to recruit host transporters within the membrane of the bacterium. However, neither of these two hypotheses has yet been demonstrated by functional studies.

The transport function of the very recently sequenced genome of *Buchnera* from another specialist aphid, *Cinara tujafilina* (closely related to *C. cedri*), is very similar to that of *BCc* [50]. Among the differences, the *fliJ*, *fliM*, *flgB*, *flgC*, *flgG*, *ychE* and *uup* genes are present in *BCt* and absent in *BCc*, whereas the *mdlA*, *mdlB*, *yoaE* and *secG* genes absent in *BCt* are present in *BCc*. The loss of mdlAB in *BCt* is consistent with the loss of primary transporters seen in the two strains, whereas the conservation of the Uup protein in *BCt* (ATP binding protein of an ABC transport system) argues in favour of an alternative role of the protein in this *Buchnera*. We noted that the flagellum is less degraded in *BCt*, compared to *BCc*.

The present analysis reveals that the selective pressures, due to the intracellular localisation of the bacteria, have shaped the transport function in *Buchnera*. In addition to the loss of diversity of the main transporter that is commonly observed in free-living bacteria, an overall lack of inner-membrane importers was observed for most of the classes of compounds that need to be exchanged between the host and the bacteria. This striking deficiency might be overcome either by the acquisition of a larger solute spectrum of the conserved transporters in *Buchnera* or, alternatively, by host-encoded transporters that could be targeted to the bacterial membranes. This “exogenous” targeting hypothesis is a classic aspect of the serial endosymbiosis theory [100]. It has been investigated for all prokaryotic-originating organelles, but it represents an interesting challenge for future research in the field of insect obligate endosymbiosis to elucidate the evolution of the association occurring between a bacterium and an already highly organized metazoan host. With the advent of genomic data, and the development of related high-throughput approaches, these questions are only just starting to be addressed in insect symbiotic models [92]. The comparative analysis performed here with *Buchnera,* from the aphids *B. pistaciae* and *C. cedri,* also revealed that the transport function could somehow be linked with the adaptive potential, the life-history traits and the specialization of aphids. At a molecular level, a functional study of the unique single membrane of *BBp*, as well as the “porous” double membrane of *BCc*, are also promising models for the future, albeit experimentally difficult.

## Supporting Information

Figure S1 **Schematics of transport capabilities in** ***Buchnera from (A)*** **Schizaphis graminum** **(** **BSg** **)**, ***(*** ***B*** ***)*** **Baizongia pistaciae** **(** **BBp** **)** ***and*** ***(*** ***C*** ***)*** **Cinara cedri** **(** **BCc** **)**. Putative transporter families are presented for each class of compounds (dotted rectangles). Transporters are coloured according to their class: primary active transporters (green), secondary transporter (blue), group translocators (yellow), channels (pink), unknown (grey). Conserved transporters in the four *Buchnera* strains are outlined with red boxes. Small encircled question mark indicates that the corresponding transporter is an hypothetical candidate showing low sequence identity with the well annotated homologous reference in TCDB and/or for which the transported substrate was not known with a high accuracy for the orthologous reference sequence in TCDB (see table 1).
(EPS)

Figure S2 **Structural analysis of bacterial (** ***Buchnera*** **) and symbiosomal membranes in** ***Cinara cedri*** **(A, B) and** ***Baizongia pistaciae*** **(C, D), and automated procedure for membrane detection.** Original pictures (upper left corner of each inlet) are filtered through FFT bandpass filter (down left corner of each inlet), regions of interest (ROI) were chosen (yellow sections) showing the membranes of *Buchnera* and the gray profiles of these ROI (upper right corner of each inlet) were displayed using two-dimensional graphs (down right corner of each inlet) of the by column average intensities of the ROI-matrix of pixels. Red dotted arrows indicate the membrane positions within each ROI (3 membranes for *Cinara cedri* and 2 membranes for *Baizongia pistaciae*).
(TIF)

Figure S3 **Ultrastructural immunogold localization of GroEL within** ***A. pisum*** **maternal bacteriocytes.** A: low magnification view of the fields analysed for GroEL label within the bacteriocytes, showing the whole bacteriocyte with its surrounding layer of sheath cells (**sc**). B: control view of the immunogold labelling, with non immune rabbit serum; C-F: views of two different magnification levels over *Buchnera* cells, showing an almost complete restriction of specific label in the electron-dense areas of *Buchnera* cytoplasm (**bcy**), no concentration of label in peripheral membrane associated regions (D, E), and very few gold granules located in bacteriocyte cytoplasmic fields (**acy**). F shows a remnant *Buchnera* (**rbuc**) cell within a multivesicular body (**mvb**, infrequent bacterial turnover in young active bacteriocytes), showing a restriction of the label in the central processed cytoplasmic area.
(TIF)

Tables S1 Essential and non-essential amino acids and derivates (Table 1), cofactors and vitamins (Table 2), input compounds (Table 3), and output compounds (Table 4) present in the *BAp* network and determination of the putative importers and exporters required for their biosynthesis. False positives (manually removed) from the list of the input (Table 5) and output (Table 6) compounds found with MetExplore.
(PDF)
