# Supplementary figures and images for: A Genomic Reappraisal of Symbiotic Function in the Aphid/Buchnera Symbiosis: Reduced Transporter Sets and Variable Membrane Organisations
Source: PLoS One. 2011 Dec 27;6(12):e29096. doi: 10.1371/journal.pone.0029096 (PMC3246468; doi:10.1371/journal.pone.0029096)

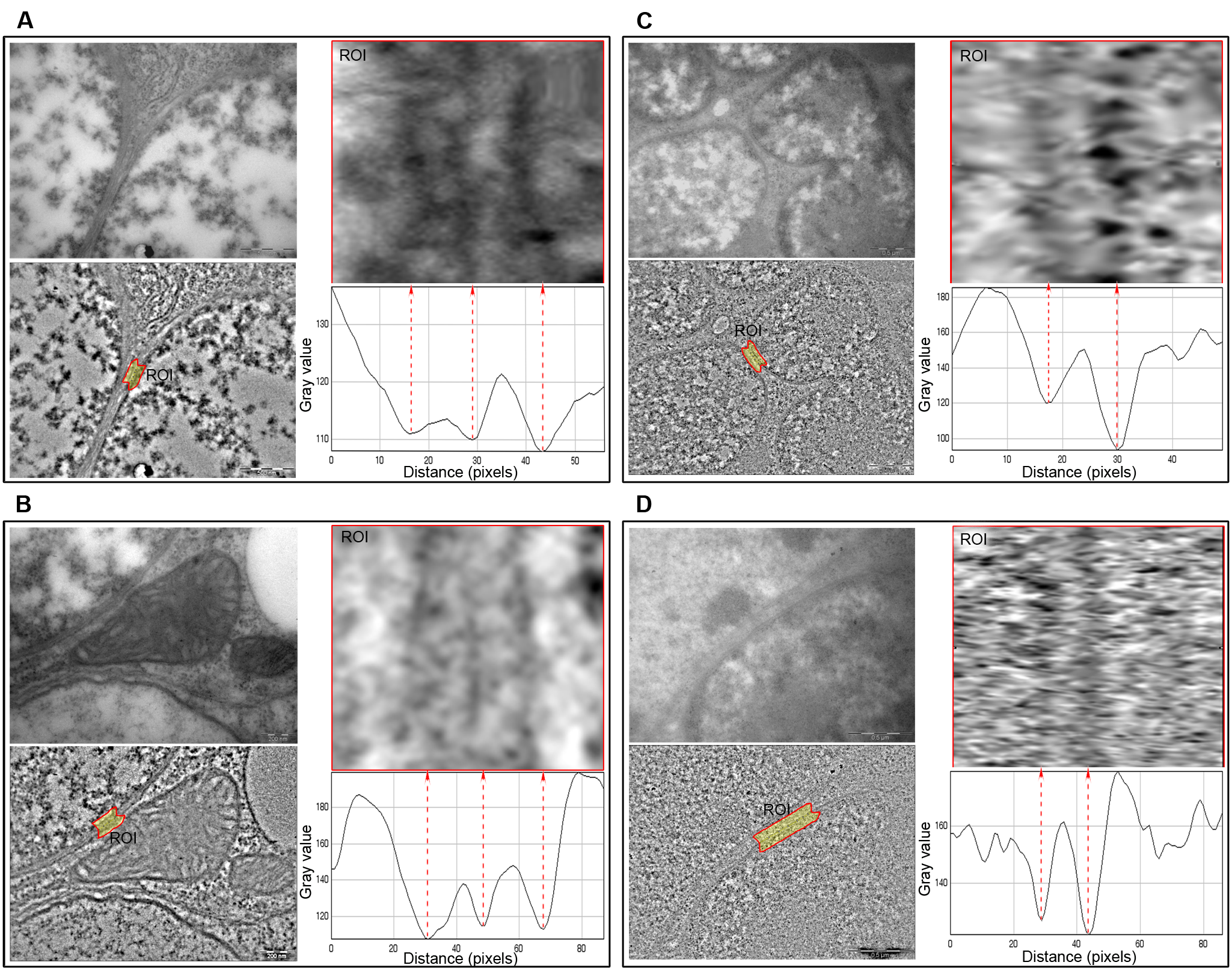

Supplement: Figure S2 — Structural analysis of bacterial ( Buchnera ) and symbiosomal membranes in Cinara cedri (A, B) and Baizongia pistaciae (C, D), and automated procedure for membrane detection. Original pictures (upper left corner of each inlet) are filtered through FFT bandpass filter (down left corner of each inlet), regions of interest (ROI) were chosen (yellow sections) showing the membranes of Buchnera and the gray profiles of these ROI (upper right corner of each inlet) were displayed using two-dimensional graphs (down right corner of each inlet) of the by column average intensities of the ROI-matrix of pixels. Red dotted arrows indicate the membrane positions within each ROI (3 membranes for Cinara cedri and 2 membranes for Baizongia pistaciae). (TIF) [file pone.0029096.s002.tif]

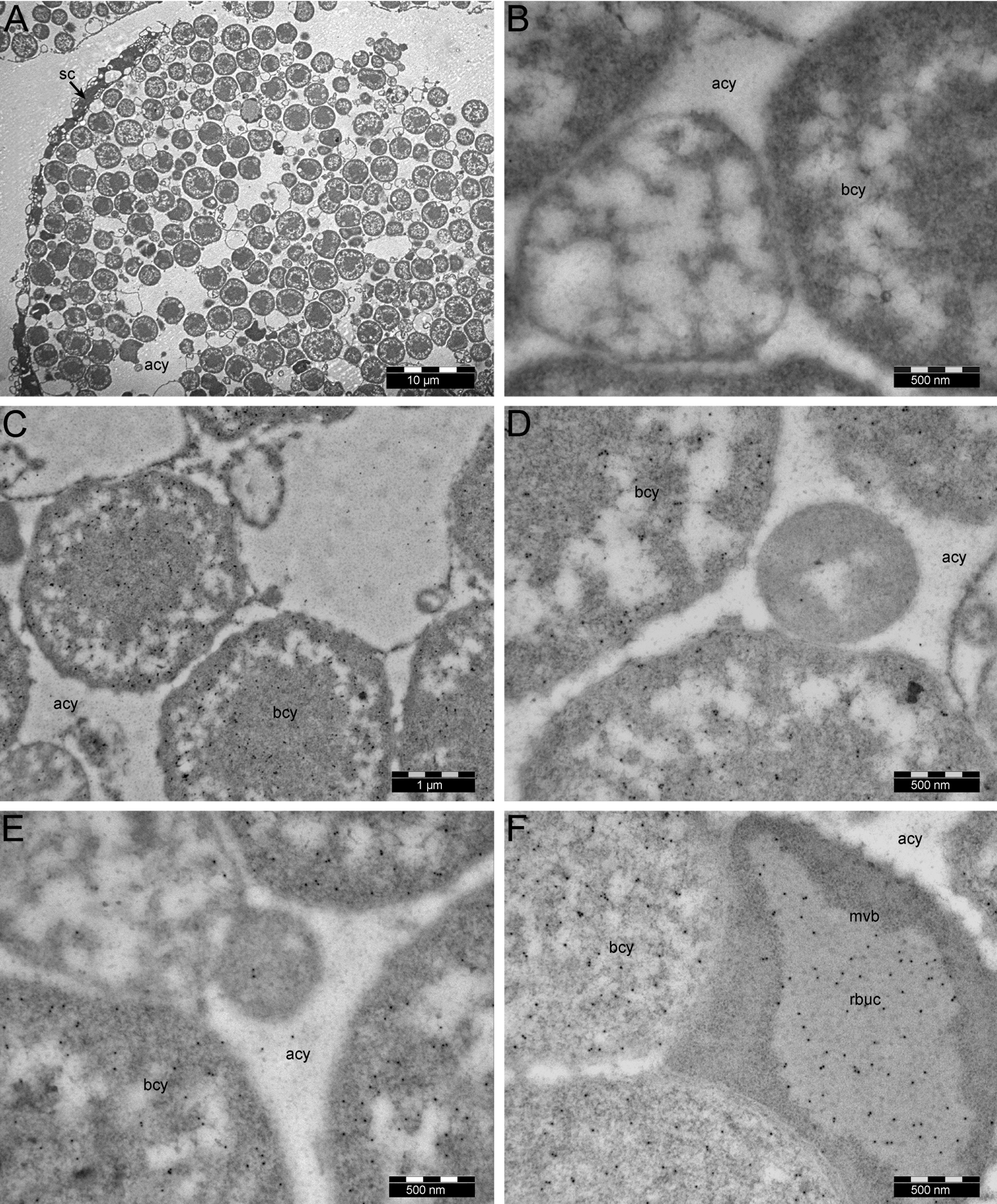

Supplement: Figure S3 — Ultrastructural immunogold localization of GroEL within A. pisum maternal bacteriocytes. A: low magnification view of the fields analysed for GroEL label within the bacteriocytes, showing the whole bacteriocyte with its surrounding layer of sheath cells (sc). B: control view of the immunogold labelling, with non immune rabbit serum; C-F: views of two different magnification levels over Buchnera cells, showing an almost complete restriction of specific label in the electron-dense areas of Buchnera cytoplasm (bcy), no concentration of label in peripheral membrane associated regions (D, E), and very few gold granules located in bacteriocyte cytoplasmic fields (acy). F shows a remnant Buchnera (rbuc) cell within a multivesicular body (mvb, infrequent bacterial turnover in young active bacteriocytes), showing a restriction of the label in the central processed cytoplasmic area. (TIF) [file pone.0029096.s003.tif]
