# Supplementary material for: A Genomic Reappraisal of Symbiotic Function in the Aphid/Buchnera Symbiosis: Reduced Transporter Sets and Variable Membrane Organisations
Source: PLoS One. 2011 Dec 27;6(12):e29096. doi: 10.1371/journal.pone.0029096 (PMC3246468; doi:10.1371/journal.pone.0029096)
Supplement: Tables S1 — Essential and non-essential amino acids and derivates (Table 1), cofactors and vitamins (Table 2), input compounds (Table 3), and output compounds (Table 4) present in the BAp network and determination of the putative importers and exporters required for their biosynthesis. False positives (manually removed) from the list of the input (Table 5) and output (Table 6) compounds found with MetExplore. (PDF) [file pone.0029096.s004.pdf]

Table 1. Essential and non-essential amino acids and derivates present in the *BAp* network and determination of the putative importers and exporters required for their biosynthesis

Amino acids	Biosynthesis in *BAp*	Required transporter
Non essential amino acids		
glutamate / glutamine	Both absent from α-ketoglutarate, present for Glu from Gln (irreversible reaction), needs at least direct import of Gln	Glu [I], Gln [I]
proline	Absent from α-ketoglutarate, needs direct import	Pro [I]
serine / glycine	Both absent from 3-phosphoglycerate, reversible interconversion is possible, needs direct import of at least one of the two	Ser [I], Gly [I]
cysteine	Absent from 3-phosphoglycerate, present from Ser, needs sulphate import and ATP	Ser [I], sulphate [I]
aspartate / asparagine	Absent from oxaloacetate, needs direct import	Asp [I], Asn [I]
alanine	Absent from aspartate and pyruvate, present from Cys (alternative pathway), needs direct import or ser import for Cys biosynthesis	Ala [I], Ser [I]
tyrosine	Absent in *Buchnera*, produced from Phe in the bacteriocyte, needs Phe or penylpyruvate export and direct import	Phe [E], Tyr [I]
Essential amino acids		
methionine	Absent from Asp, present from homocystéine and THF	Cys [I], homocysteine [I], THF [I]
threonine	Complete from Asp	Asp [I]
lysine	Complete from Asp and Glu	Asp [I], Glu [I]
valine	Incomplete from glucose (pyruvate), needs export of 2-ketoisovalerate for final transamination in the bacteriocyte and direct import.	glucose [I], 2-ketoisovalerate [E], Val [I]
leucine	Incomplete from glucose (pyruvate), needs export of 2-ketoisocaproate for final transamination in the bacteriocyte and direct import.	glucose [I], 2-ketoisocaproate [E], Leu [I]
isoleucine	Incomplete from Asp (Thr), needs export of Thr, import of 2-oxobutanoate, export of 2-keto-3-methyl-valerate for final transamination in the bacteriocyte and direct import.	Asp [I], Thr [E], 2-oxobutanoate [I], 2-keto-3-methyl-valerate [E], ile [I]
tryptophan / phenylalanine	Complete from glucose (chorismate), needs Ser and Gln (for Trp), phenylpyruvate (for Phe) is probably exported for final transamination in the bacteriocyte	glucose [I], Ser [I], Gln [I], phenylpyruvate [E]
histidine	Complete from glucose (ribose 5-P), needs Gln	glucose [I], Gln [I]
arginine	Complete from Glu and Asp, needs Glu and Asp	Glu [I], Asp [I]
Derivates		
homocysteine	Incomplete from sulfite, needs direct import	Cys [E], homocysteine [I]
ornithine	Complete in the Arg pathway, might be imported however (Poliakov et al. 2011)	Ornithine [I]

Table 2. List of cofactors and vitamins present in the *BAp* network and determination of the putative importers and exporters required for their biosynthesis

Cofactors / vitamins	Biosynthesis pathway	Required transporters
AMP , ADP, ATP, UDP, UTP, GDP, GTP, CMP, CDP, CTP, dADP, dATP	Incomplete, needs to import guanosine, inosine and putrescine, putative export of adenine	guanosine [I], inosine [I], putrescine [I], adenine [E]
biotin (vitamin B7)	Incomplete, needs malonyl-CoA (if the pathway is complete) or 7-keto-8-aminopelargonate (although not produced by the aphid), putative export for the host	malonyl-CoA [I], 7-keto-8-aminopelargonate [I], biotin [E]
CO-A, pantothenate (vitamin B5)	Incomplete, from panthoténate (vitamin B5) needs direct import or import of pantetheine phosphate	CoA [I], pantetheine 4'-P [I]
cobalamin (vitamin B12)	Absent, used as cofactor by cysG only (HAMAP), needs direct import	cobalamin [I]
Cyt-C (cytochrome C)	Incomplete, needs direct import or import of uroporphyrinogen III and protohaem	Cyt C [I], uroporphyrinogen III [I], protohaem [I]
DHF, THF (dihydro- and terahydro-folate, vitamin B9)	Incomplete from GTP and chorismate, needs direct import or import of dihydropteroate	DHF [I], 7-8,dihydropteroate [I], formaldehyde [I]
FAD, FADH2, FMN, FMNH2 (vitamin B2)	Almost Complete from GTP and ribulose 5-P, putative export for the host	riboflavin [E],
gluthatione	Present from Glu and Cys (Cys is produced from Ser and Sulphate), needs Gly, probably exported (Poliakov et al. 2011)	Glu [I], Ser [I], sulphate [I], Gly [I], glutathione [E]
niacine, NAD, NADH, NADPH (vitamin B3)	Incomplete, needs import of nicotinate (vitamin B3)	nicotinate [I]
pyridoxine (vitamin B6)	Absent, pyridoxal used by > 5 enzymes (HAMAP), needs direct import	pyridoxine [I]
SAM, SAH	Complete from methionine, needs import of homocysteine and export of cysteine	homocysteine [I], Cys [E]
thiamine (vitamin B1)	Absent, used by >5 enzymes (HAMAP), needs direct import	thiamine [I]
ubiquinol, ubiquinone	Absent, needs direct import	ubiquinol [I], ubiquinone [I]

Table 3. List of the input compounds (precursors) from the metabolic network of *Bap,* found with MetExplore.

**Input compound**	**Main biosynthesis pathways**	**TI** [1]
amino acids (see Table 3)		
Vitamins and cofactors (see Table 3)		
2-oxobutanoate	Ile	[a]
7,8-dihydropteroate	THF	
α-D-glucose 6-phosphate	Central metabolism	
formaldehyde	Methyl donor (especially useful if THF is imported rather than 5,10-methylene THF	
inosine	Pyrimidine biosynthesis	
L-1-phosphatidyl-glycerol	Cardiolipin biosynthesis	
7-keto-8-aminopelargonate	Biotin biosynthesis (not produced by *A. pisum*). Malonyl-CoA might be the true precursor	
Mannitol-1P	Central metabolism	
pantetheine 4'-P	Biosynthesis of CoA	
putrescine	Biosynthesis of purines and pyrimidines	
uroporphyrinogen and protoheme	Biosynthesis of CytC	

[1] Topological Information: [a] Source, the metabolite is not produced by any reaction and is consumed by one reaction; (from MetExplore, http://metexplore.toulouse.inra.fr).

Table 4. List of the output compounds from the metabolic network of *Bap,* found with MetExplore.

Output compound	Main reaction	TI[1]
Amino acids (see Table 4)		
Vitamins and cofactors (see Table 4)		
2-keto-3-methyl-valerate	Ile biosynthesis, the transamination is occurring in the bacteriocyte	[a]
2-ketoisocaproate	Leu biosynthesis, the transamination is occurring in the bacteriocyte	
2-ketoisovalerate	Val biosynthesis, the transamination is occurring in the bacteriocyte	
ADP-D-glycero-D-manno-heptose	Compound used for the LPS biosynthesis (the last step with the racemase is lacking) and the biosynthesis of the lipid part is not known	
biotin	biotin biosynthesis	
FAD (flavin adenine dinucleotide)	flavin and derivates biosynthesis	
FMNH2 (reduced flavin mononucleotide)	flavin and derivates biosynthesis	[b]
fumarate	Arg biosynthesis, as the TCA cycle is non functional fumarate might accumulate and might be exported	
glutathione	might need export as the salvage pathway is absent	
glycerol	Produced in the cardiolopine biosynthesis pathway	
phenylpyruvate	Phe biosynthesis, the transamination is occurring in the bacteriocyte	
spermidine	Purine and pyrimidine pathway, might accumulate with no possibility of salvage	
succinate	linked with fumarate, as the TCA cycle is non functional it might accumulate	

[1] Topological information: [a] Dead End Choke Point, the metabolite is uniquely produced by a specific reaction and not consumed by any reaction; [b] Dead End, the metabolite is not consumed by any reaction and is produced by several reactions (from MetExplore, http://metexplore.toulouse.inra.fr).

Table 5. False positives (manually removed) from the list of the input compounds found with MetExplore.

Input compound	Comment about manual removing	TI[1]
(2S)-2-amino-3-oxo-4-phosphonooxybutanoate	Not relevant as the corresponding pathway is incomplete (correspond to a putative spontaneous reaction)	a
2-oxo-3-hydroxy-4-phosphobutanoate	Pyridoxal 5-P biosynthesis, the reaction pointed for SerC is probably not relevant here.	
3-phospho-hydroxypyruvate	Ser biosynthesis, probably not used as serine is imported in the cell	
β-alanine	Probably not used as CoA is directly imported	
cadaverine	Probably not used, as metabolism is oriented towards spermidine rather than aminopropyl-cadaverine	
coproporphyrinogen III	Probably not imported as uroporphyrinogen-III is imported	
CPD-689, cob(II)yrinate a,c-diamide	Probably not used in the network as cobalamine is imported	
CPD-9451, isopropylmaleate	Probably not used as the flux is oriented toward leucine production	b
CPD-1302, 5-methyltetrahydropteroyltri-L-glutamate	Another methyl donor is probably used	
CPD-5727, 5,10-methenyl-tetrahydropteroyl-[γ-Glu](n)	Not used in the network, the enzyme FolD is also the methylene-THF desydrogenase	
D-alanyl-D-alanine	Peptidoglycan biosynthesis - the ddlB might be functional in *BAp* (Poliakov et al. 2011)	
D-myo-inositol (3)-monophosphate	Probably not used (myo-inositol pathway), the phosphatase *suhB* is probably active for other processes	
glutamate-1-semialdehyde	The reaction pointed for ArgD is probably not relevant here (tetrapyrrole biosynthesis)	
L-pantoate	Probably not used as panthotenate (or coA) is directly imported	
N2acetyl-α-aminoadipyl-δ-phosphate	The role of ArgC in the biosynthesis pathway of the Lys is not relevant	
porphobilinogen	Probably not imported as uroporphyrinogen-III is imported	
propionyl-CoA	The enzymes Pta and AckA are probably not used in this incomplete patway (thr degradation)	
5-amino-6-(5-phospho-ribitylamino)-uracil	One step (hydrolase/phosphorilase) lacking in the riboflavine biosynthesis. The shuttle may not be possible for such a reaction that is probably occurring with another enzyme in *Buchnera*.	
SAICAR, 5'-phosphoribosyl-4-(N-succinocarboxamide)-5-aminoimidazole	Probably not used as import of inosine and adenine should initiate the pathways	
S-lactoyl-glutathione	The enzyme Glob is probably not relevant for this reaction as lactate is not involved in the network of *B. aphidicola*	
xanthine	The reaction pointed for Gpt is probably not relevant as salvage and biosynthesis of purine are altered	

[1] Topological Information: [a] Source, the metabolite is not produced by any reaction and is consumed by several reactions; [b] Source or Dead End Choke Point, the metabolite is uniquely consumed or produced by the same reversible reaction (from MetExplore, http://metexplore.toulouse.inra.fr).

Table 6. False positives (manually removed) from the list of the output compounds found with MetExplore.

Output compound	Comment about manual removing	TI[1]
1-amino-propan-2-one-3-phosphate	Not produced (artefact linked to a spontaneous reaction in the pyridoxal 5-P biosynthesis pathway)	[a]
2-dehydropantoate	Probably not produced in the network as coA is imported	
3-phospho-serine	Probably not produced in the network as Ser is imported	
4-(phosphonooxy)-threonine	Probably not produced (the transaminase SerC is not active on this process)	
5-amino-levulinate	The enzyme ArgD is not relevant for this degenerated pathway (tetrapyrrole biosynthesis)	
cardiolipin	Probably not accumulated (salvage through reversible reaction)	
CPD-694 (cob(I)yrinate a,c-diamide)	Probably not produced in the network as cobalamine is imported	
CPD-1086 (5-amino-6-(5'-phosphoribitylamino)uracil)	The compound is probably not accumulated nor exported, the dephosphorylation is probably occurring in the cell by an unknown phosphatase	
CPD-1301, tetrahydropteroyltri-L-glu	Probably not produced as folate, is imported in the cell	
deoxyribose-5-phosphate	Probably not accumulated (reversible reaction)	
CPD0-1065, aminopropylcadaverine	Probably not produced in the network as the flux is oriented towards spermidine	
D-lactate	Probably not accumulated as the transformation lactate to pyruvate is feasible	
erythronate-4-phosphate	Probably not produced by GapA in this degenerated pathway (pyridoxal biosynthesis)	
hydroxymethylbilane	Probably not produced as protohaem is imported in the cell	
myo-inositol	Probably not used, the phosphatase *suhB* is probably active for other processes	
N2-acetyl-α-aminoadipate semialdehyde	The role of ArgC in the biosynthesis pathway of the Lys is not relevant	
PAPS, phosphoadenosine-5'-phosphosulfate	Probably not produced in the network as coA is imported	
protoporphyrinogen IX	Involved in the Met/SAM cycle (probably not accumulated)	
S-adenosyl-4-methylthio-2-oxobutanoate	biotin and methionine biosynthesis, might not accumulate with possibility of salvage (SAM + 2-oxobutanoate)	
S-methyl-5'-thioadenosine	Probably not produced in the network as coA is imported	
sirohaem	Probably not accumulated (however the salvage is not described in BioCyc and KEGG)	
thiamin diphosphate	The reaction is not occurring as thiamin is imported	
UDP-N-acetylmuramoyl-tripeptide	Probably not exported, D-ala should be imported and the 6.3.2.10 reaction may happen although the corresponding enzyme is not known	
xanthosine-5-phosphate	Probably not produced as the purine salvage is degraded	

[1] Topological Information: [a] Source, the metabolite is not produced by any reaction and is consumed by several reactions (from MetExplore, http://metexplore.toulouse.inra.fr).
